# Hepatic Sirt6 activation abrogates acute liver failure

**DOI:** 10.1038/s41419-024-06537-5

**Published:** 2024-04-22

**Authors:** Jinque Luo, Huan Liu, Yanni Xu, Nanhui Yu, Rebbeca A. Steiner, Xiaoqian Wu, Shuyi Si, Zheng Gen Jin

**Affiliations:** 1 Aab Cardiovascular Research Institute, Department of Medicine, University of Rochester School of Medicine and Dentistry, 601 Elmwood Avenue, Box CVRI, Rochester, NY 14642 USA; 2 Institute of Medicinal Biotechnology, Chinese Academy of Medical Sciences and Peking Union Medical College (CAMS & PUMC), No. 1 Tiantan Xili, Beijing, 100050 China; 3 Hunan Provincial Key Laboratory of the Research and Development of Novel Pharmaceutical Preparations, “The 14th Five-Year Plan” Application Characteristic Discipline of Hunan Province (Pharmaceutical Science), College of Pharmacy, Changsha Medical University, Changsha, 410219 Hunan China; 4 State Key Laboratory of Experimental Hematology, National Clinical Research Center for Blood Diseases, Haihe Laboratory of Cell Ecosystem, Institute of Hematology & Blood Diseases Hospital, Chinese Academy of Medical Sciences & Peking Union Medical College, Tianjin, 300020 China; 5 The 2nd Xiangya Hospital, Central South University, Changsha, 410011 Hunan China; 6 Guangzhou Municipal and Guangdong Provincial Key Laboratory of Molecular Target & Clinical Pharmacology, the NMPA Key Laboratory of Respiratory Disease, School of Pharmaceutical Science, Guangzhou Medical University, Guangzhou, China

**Keywords:** Necroptosis, Hepatotoxicity

## Abstract

Acute liver failure (ALF) is a deadly illness due to insufficient detoxification in liver induced by drugs, toxins, and other etiologies, and the effective treatment for ALF is very limited. Among the drug-induced ALF, acetaminophen (APAP) overdose is the most common cause. However, the molecular mechanisms underlying APAP hepatoxicity remain incompletely understood. Sirtuin 6 (Sirt6) is a stress responsive protein deacetylase and plays an important role in regulation of DNA repair, genomic stability, oxidative stress, and inflammation. Here, we report that genetic and pharmacological activation of Sirt6 protects against ALF in mice. We first observed that Sirt6 expression was significantly reduced in the liver tissues of human patients with ALF and mice treated with an overdose of APAP. Then we developed an inducible Sirt6 transgenic mice for Cre-mediated overexpression of the human Sirt6 gene in systemic (Sirt6-Tg) and hepatic-specific (Sirt6-HepTg) manners. Both Sirt6-Tg mice and Sirt6-HepTg mice exhibited the significant protection against APAP hepatoxicity. In contrast, hepatic-specific Sirt6 knockout mice exaggerated APAP-induced liver damages. Mechanistically, Sirt6 attenuated APAP-induced hepatocyte necrosis and apoptosis through downregulation of oxidative stress, inflammation, the stress-activated kinase JNK activation, and apoptotic caspase activation. Moreover, Sirt6 negatively modulated the level and activity of poly (ADP-ribose) polymerase 1 (PARP1) in APAP-treated mouse liver tissues. Importantly, the specific Sirt6 activator MDL-800 exhibited better therapeutic potential for APAP hepatoxicity than the current drug acetylcysteine. Furthermore, in the model of bile duct ligation induced ALF, hepatic Sirt6-KO exacerbated, but Sirt6-HepTg mitigated liver damage. Collectively, our results demonstrate that Sirt6 protects against ALF and suggest that targeting Sirt6 activation could be a new therapeutic strategy to alleviate ALF.

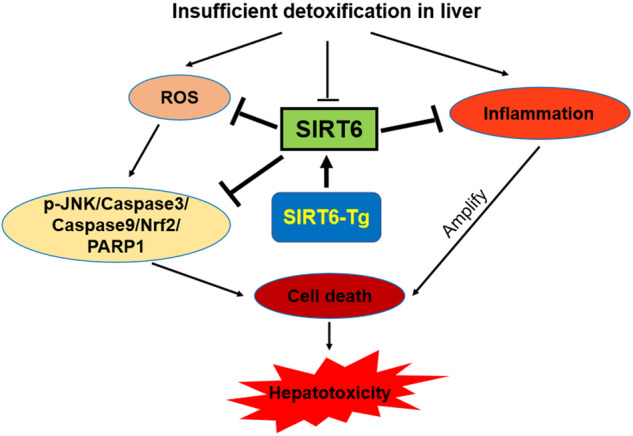

## Introduction

Acute liver failure (ALF) is a life-threatening condition with abrupt hepatocyte injury and rapid deterioration of liver function, which can evolve to lethal outcome [1, 2]. Manifests with a variety of insults to liver cells, a consistent pattern of rapid-onset impaired coagulation, elevation of aminotransferases, and alteration of mental status, ALF is seen presents unique challenges in clinical management and carries a very high mortality. Causes of ALF include overdose of medicine, hepatic ischemia, herbal and dietary poisoning, and autoimmune hepatitis [3–5]. Among these, medicine overdose remains most frequent cause of ALF in United States of America and many other countries [6]. Most medicine-induced ALF occurs due to either intentional overdose or unintentional ingestion of acetaminophen (APAP). APAP is the commonly used component for pain and fever, can found in many over-the-counter and prescription medicines. In most cases, therapeutic dose of APAP is non-toxic, while an overdose leads to substantial liver injury [7, 8]. The mechanisms of APAP-induced liver injury remain not fully understood, although necrosis and apoptosis have been proposed to play a pivotal role [9, 10]. APAP was metabolized in hepatocytes mainly through the cytochrome P450s, such as CYP2E1, CYP1A2, and CYP3A11 [11, 12]. The toxicity of APAP stems from its metabolite, N-acetyl-p-benzoquinone imine (NAPQI), which could form covalent bonds with intracellular proteins, induce the formation of APAP-adduct, and eventually exert harmful effects on liver function [13, 14].

Due to the dose-dependent toxicity, APAP-induced liver failure is a highly fidelity and adjustable model that most mechanisms are translatable to humans [15]. Except this, there are a number of models have developed that approximate the clinical ALF in patients, such as bile duct ligation (BDL) and hepatic ischemia-reperfusion injury [16]. BDL is a commonly used surgical method to induce cholestatic ALF in animal models [17]. Cholestasis induced by surgical ligation of the common bile duct led to typical time-dependent morphological and structural changes in the liver, result in liver damage within 5–7 days. All these models are imperative to our understanding of the associated human conditions and finding new and effect approaches to patient care.

Sirtuin 6 (Sirt6), an important member of highly conserved Sirtuin family, is a well-studied NAD^+^-dependent histone deacetylase. Sirt6 has been implicated in the regulation of aging, inflammation, and genome stability [18–20]. Accumulating data suggest that Sirt6 may serve as a therapeutic target for liver disease such as fatty liver, liver steatosis, and alcohol-induced tissue injury [21, 22]. Hepatocyte-specific Sirt6 knockout mice manifested liver fibrosis through the regulation of c-JUN signaling [18]. Sirt6 overexpression reduces inflammatory response evidenced by suppressing multiple inflammatory mediators [23, 24]. It has been reported that Sirt6 knockdown exaggerated APAP-induced hepatocytes damage [25]. However, the potential protective effects of Sirt6 transgenic overexpression in vivo on APAP- or BDL-induced ALF remain to be investigated.

In this study, we investigated the roles of Sirt6 in ALF. We first examined the levels of Sirt6 expression in the liver tissues of human patients with ALF and mice treated with an overdose of APAP. We then generated and utilized global and hepatocyte-specific human Sirt6 transgenic (Sirt6-Tg and Sirt6-HepTg) mice and hepatocyte-specific Sirt6 knockout (Sirt6-HepKO) mice, the oral treatment of pharmacological Sirt6 activator, and the cultured primary hepatocytes. We uncovered that Sirt6 protected against APAP-induced liver injury by alleviating hepatic cell death. Moreover, we found that Sirt6 also exhibited a protective role for BDL-induced ALF. Our studies described herein reveal that Sirt6 could be a potential therapeutic target for the treatment of ALF.

## Results

### Sirt6 expression was decreased in the liver tissues of patients with hepatic failure and APAP overdose-treated mice

**Fig. 1:**
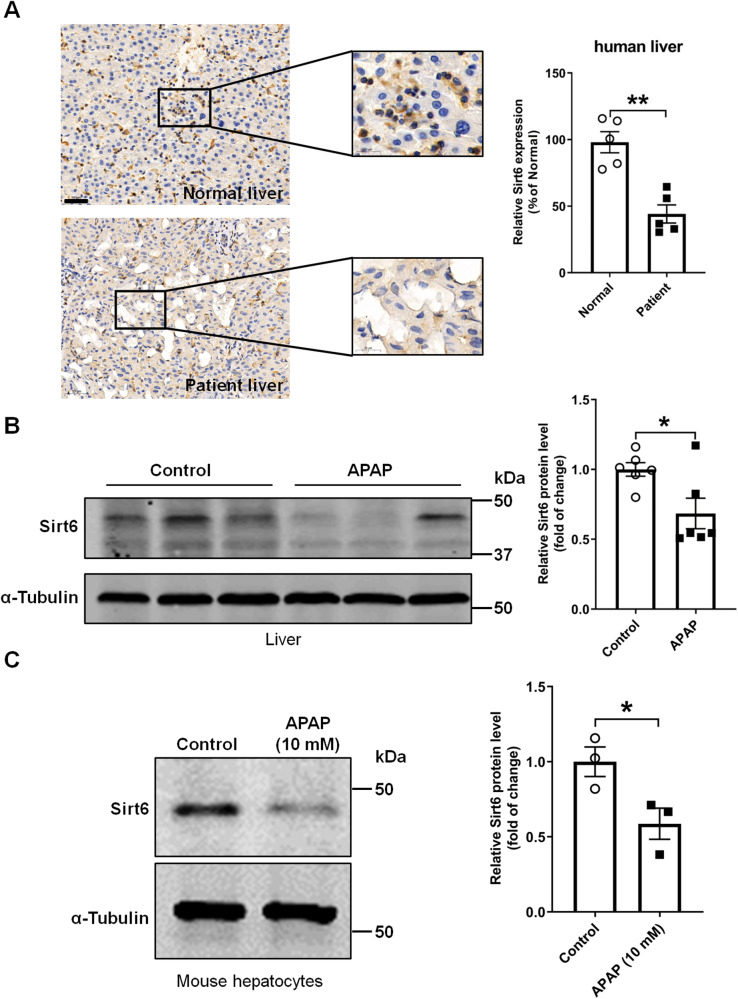
Sirt6 was decreased in the livers of hepatic failure patients and APAP treated mice. **A** Representative anti-Sirt6 immunostaining in human liver sections from healthy individual and liver failure patients who required liver transplantation. Scale bar corresponds to 50 μM. Sirt6 express levels via immunostaining were quantified. Results were expressed as relative change to the Normal group, and data were presented as mean ± SEM (*n* = 5). ^**^P < 0.01. **B** Sirt6 protein levels in the liver tissues from WT mice i.p. injected 500 mg/kg APAP or saline for 8 h. Blots were quantified and results were expressed as fold change relative to the saline-injected condition (Control group), and data were presented as mean ± SEM (*n* = 6, each lane represents a different mouse). ^*^P < 0.05. **C** Western blotting of Sirt6 in normal C57BL/6J mouse hepatocytes treated with 10 mM of APAP for 8 h. Blots were quantified and results were expressed as fold change relative to the vehicle-treatment condition (Control group), and data were presented as mean ± SEM (*n* = 3). ^*^P < 0.05. Statistical analysis was performed by unpaired Student’s *t* test.

To assess the relevance of Sirt6 to human liver failure, we analyzed Sirt6 expression using immunohistochemistry analysis in hepatic tissues from individuals with disease-associated liver failure who required liver transplantation. As shown in Fig. 1A, Sirt6 levels were significantly decreased in the liver of hepatic failure patients compared to those from healthy subjects.

To investigate the relevance of hepatic Sirt6 to APAP-induced liver injury, we analyzed Sirt6 expression in the liver tissues from C57BL/6J mice that fasted overnight and treated with 500 mg/kg of APAP by intraperitoneal injection. As depicted in the Western blotting analysis of Fig. 1B, APAP treatment for 8 h led to a marked decrease of Sirt6 protein level in the liver of C57BL/6J mice. Moreover, we isolated primary hepatocytes from C57BL/6J mice to further evaluate the effect of APAP on Sirt6 expression, APAP (10 mM) treatment for 8 h significantly decreased the protein level of Sirt6 in primary hepatocytes (Fig. 1C). These results indicated that the downregulation of Sirt6 is implicated in both human and mice with acute liver failure.

### Sirt6 overexpression suppressed APAP-induced hepatotoxicity in mice

**Fig. 2:**
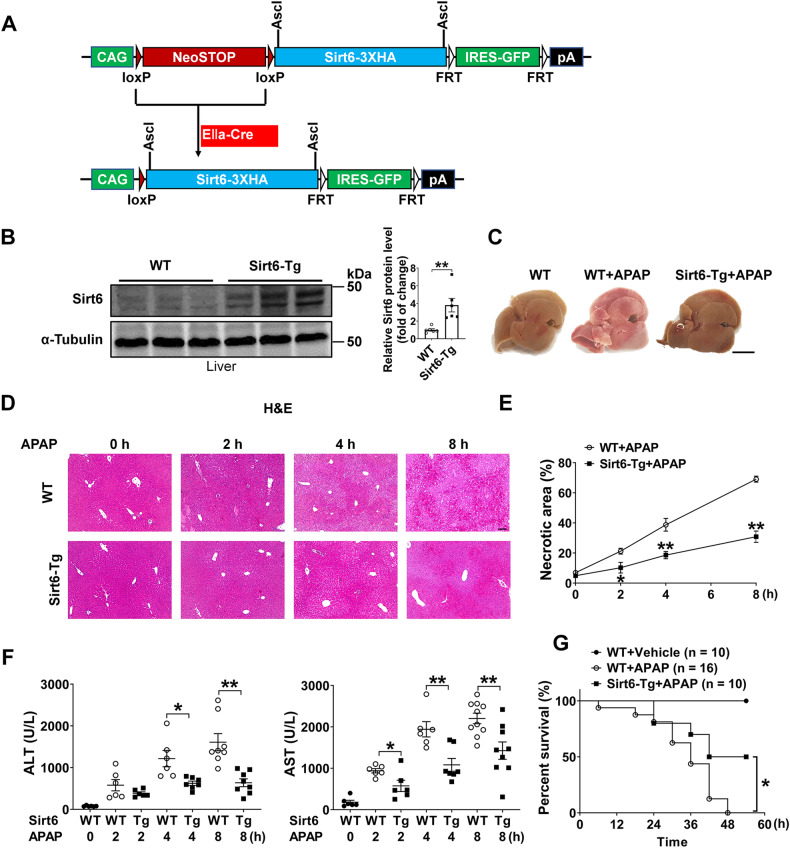
Sirt6 overexpression suppressed APAP-induced hepatotoxicity. **A** Schematic diagram for the generation of the Sirt6 transgenic Sirt6-Tg mice. **B** Sirt6 protein levels were detected in the liver tissues derived from WT or Sirt6-Tg mice without APAP or saline injection. Blots were quantified and results were expressed as fold change relative to WT mice. Results were presented as mean ± SEM (*n* = 6), **P < 0.01. **C** WT and Sirt6-Tg mice were treated with APAP (500 mg/kg) or saline for 8 h and then the liver tissues were harvested. Representative images showed the appearance of mouse livers. Scale bar corresponds to 5 mm. **D**, **E** Representative images of H&E-stained liver sections from WT and Sirt6-Tg mice treated with APAP (500 mg/kg) or saline for the indicated times. 10×, scale bar corresponds to 200 µm. Quantification of necrotic areas by ImageJ software. Results were presented as mean ± SEM (*n* = 3), *P < 0.05, **P < 0.01 compared with the WT + APAP group. **F** Serum ALT (left) and AST (right) activity in liver tissues from WT and Sirt6-Tg mice treated with APAP (500 mg/kg) or saline for the indicated times. Each dot or triangle symbol represents a different mouse sample. Results were presented as mean ± SEM (*n* = 6–10 for each genotype at each time point), *P < 0.05, **P < 0.01. **G** Survival rate of WT and Sirt6-Tg mice treated with APAP (750 mg/kg) for 54 h, *P < 0.05. Statistical analysis was performed by unpaired Student’s *t* test (**B**), two-way ANOVA plus Bonferroni’s multiple comparisons test (**E**), one-way ANOVA plus Dunnett’s multiple comparisons test (**F**), and log-rank test (**G**).

To ask whether Sirt6 has a protective role in APAP-induced liver injury, we generated global Sirt6 transgenic (Sirt6-Tg) mice in which human Sirt6 was conditionally induced under the cytomegalovirus enhancer fused to the chicken *β-actin* (CAG) promoter (Fig. 2A). The level of Sirt6 protein was significantly increased in the Sirt6-Tg mouse livers (Fig. 2B). Both the wild type (WT) mice and Sirt6-Tg mice were administered with 500 mg/kg of APAP. After 8 h, the mice were sacrificed and tested for the markers of liver damage. Results showed that Sirt6 overexpression significantly protected mice from APAP-induced liver injury. Specifically, histological analysis revealed that there was an abundant presence of necrotic areas in the livers of WT mice at 2 h after APAP administration, while much less centrilobular necrotic lesions in Sirt6-Tg mice were observed compared with those in WT mice (Fig. 2C–E). Protection of liver damage in Sirt6-Tg mice was further determined by the analysis of ALT and AST in serum, which are commonly used in the clinic to diagnose ALF. Results revealed markedly lower serum levels of ALT in Sirt6-Tg mice than that in the WT group after APAP administration (625.9 ± 51.63 vs 1217 ± 196 at 4 h, 638.6 ± 94.18 vs 1610 ± 205.2 at 8 h, means ± SEM), similar outcomes are seen in AST levels between APAP treated Sirt6-Tg group and WT group (575.3 ± 137 vs 940.9 ± 52.46 at 2 h, 1084 ± 153.8 vs 1942 ± 184.6 at 4 h, 1427 ± 209.2 vs 2204 ± 128 at 8 h, means ± SEM) (Fig. 2F). Furthermore, we used 750 mg/kg APAP to challenge the mice and determine the survival rate, the results showed that Sirt6-Tg also improved survival of mice within 54 h (Fig. 2G). These results revealed a critical protective role of Sirt6 gain-of-function in APAP-induced hepatotoxicity.

### Sirt6 overexpression counteracted APAP-induced hepatocyte death

**Fig. 3:**
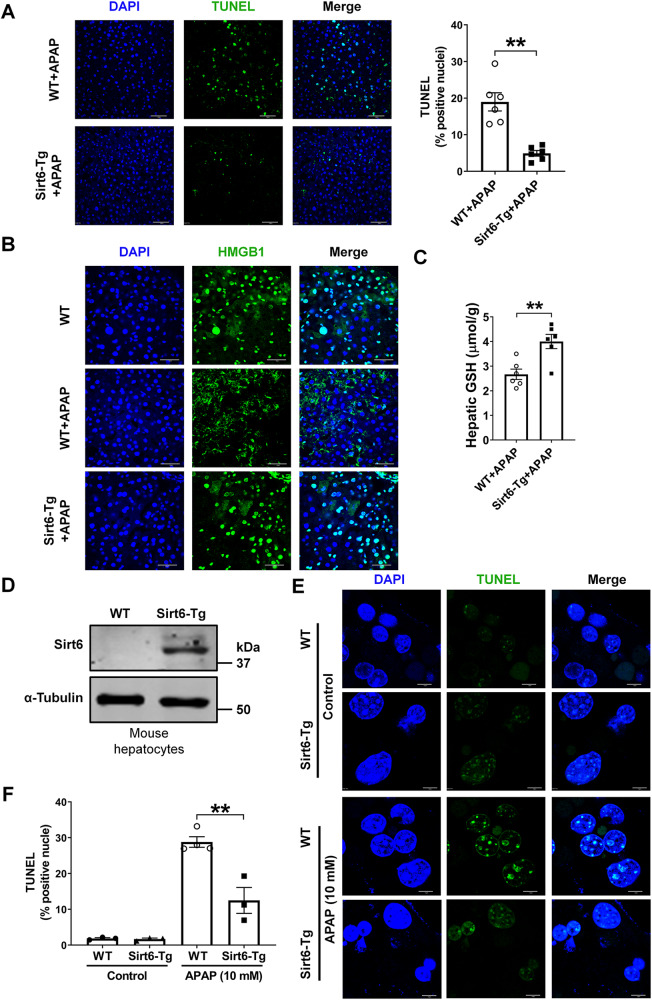
Sirt6 overexpression counteracted APAP-induced hepatocyte death. WT and Sirt6-Tg mice were treated with APAP (500 mg/kg) for 8 h. Liver sections were prepared and analyzed for the markers of cell death. **A** TUNEL staining of the liver sections. scale bar corresponds to 50 μm, and results were presented as mean ± SEM (*n* = 6), ^**^P < 0.01. **B** HMGB1 staining of liver sections, scale bar corresponds to 40 μm. **C** Liver GSH concentrations. Results were presented as mean ± SEM (*n* = 6), ^**^P < 0.01. **D** Western blotting of Sirt6 in mouse hepatocytes isolated from WT mice and Sirt6-Tg mice. **E**, **F** TUNEL assay of cell death of WT and Sirt6-Tg mouse hepatocytes treated with APAP (10 mM) or PBS for 8 h. Scale bar corresponds to 10 μm. Results were presented as mean ± SEM (*n* = 3–4), ^**^P < 0.01. Statistical analysis was performed by unpaired Student’s *t* test (**A,** **C**), and two-way ANOVA plus Bonferroni’s multiple comparisons test (**F**).

After metabolizing into NAPQI, APAP could lead to hepatic GSH depletion, hepatocyte nuclear DNA fragmentation, and eventually necrotic cell death. TUNEL assay demonstrated lower frequency of nuclear DNA fragmentation in Sirt6-Tg mice compared with WT mice (Fig. 3A). The binding chromatin nuclear protein HMGB1 is a molecule usually used to characterize the necrosis, and immunostaining results showed that HMGB1 exerted cytoplasmic translocation in the liver tissues from APAP-induced WT mice, whereas Sirt6 overexpression nullified the HMGB1 cytoplasmic translocation and inhibited the HMGB1 expression in mouse liver (Fig. 3B). Taking into account that APAP-induced liver injury is highly GSH dependent, we next determined GSH level in wild-type and Sirt6-Tg mice following APAP administration, as show in Fig. 3C, GSH level was significant higher in Sirt6-Tg liver compared to values of the WT controls. These results suggested that Sirt6 alleviates APAP-induced liver injury by decreasing hepatocyte necrosis.

To further verify the protective effect of Sirt6 overexpression on APAP-induced hepatocytes damage, we isolated primary hepatocytes from WT and Sirt6-Tg mice and treated them with APAP. Expression of Sirt6 protein was markedly higher in the hepatocytes from the Sirt6-Tg mice hepatocytes than that in the hepatocytes from WT mice (Fig. 3D). TUNEL assay showed that marked toxicity in WT primary hepatocytes after APAP (10 mM) treatment, but hepatocytes from Sirt6-Tg mice were significantly protected (Fig. 3E, F). These results suggested that Sirt6 overexpression ameliorates APAP-induced acute hepatocytes death.

### Sirt6 overexpression suppressed APAP-induced JNK activation in mouse liver tissues

We then investigated the molecular mechanism by which Sirt6 overexpression protects against liver injury and hepatocyte death in response to APAP treatment. Since CYP2E1, CYP1A2, and CYP3A11 are the most active P450s enzymes that convert APAP to NAPQI. We first measured the mRNA and protein levels of these three enzymes. The results indicated that no alterations in the levels of APAP-metabolizing enzymes were evident between Sirt6-Tg mice and WT mice. The liver NAPQI was also not altered in Sirt6-Tg mice compared with WT mice (Fig. S1), suggesting that Sirt6 has minimal impact on the hepatic metabolism of APAP.

**Fig. 4:**
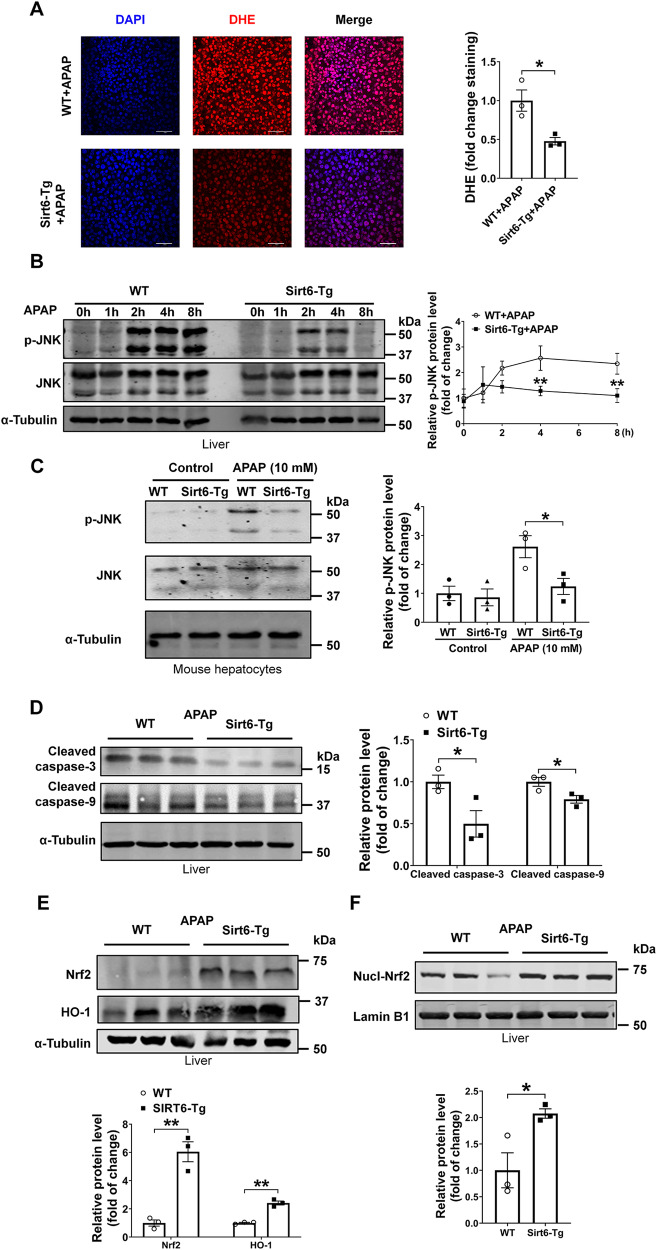
Sirt6 overexpression reduced APAP-induced liver necrosis and apoptosis. **A** The production of ROS in vivo was measured by DHE staining in the liver sections of WT and Sirt6-Tg mice treated with APAP (500 mg/kg) for 8 h (scale bar corresponds to 50 μm). Results were presented as mean ± SEM (*n* = 3), ^*^P < 0.05. **B** JNK activation by Western blotting in liver extracts from WT and Sirt6-Tg mice treated with APAP (500 mg/kg) or saline for the indicated times. Blots were quantified and results were presented as mean ± SEM (*n* = 5 for each genotype at each time point), ^**^P < 0.01 compared with the WT + APAP group. **C** JNK activation analyzed by Western blotting in WT and Sirt6-Tg mouse hepatocytes treated with APAP (10 mM) or PBS for 8 h. Blots were quantified and results were presented as mean ± SEM (*n* = 3), ^*^P < 0.05. **D** Western blotting of indicated proteins in the liver tissues from WT and Sirt6-Tg mice treated with APAP (500 mg/kg) for 8 h. Blots were quantified and results were presented as mean ± SEM (*n* = 3), ^*^P < 0.05. **E** Western blotting of Nrf2, HO-1 in the liver tissues from WT and Sirt6-Tg mice treated with APAP (500 mg/kg) for 8 h. Blots were quantified and results were presented as mean ± SEM (n = 3), ^**^P < 0.01. **F** Nrf2 nuclear translocation in the liver tissues from WT and Sirt6-Tg mice treated with APAP (500 mg/kg) for 8 h. Blots were quantified and results were presented as mean ± SEM (*n* = 3), *P < 0.05. Statistical analysis was performed by unpaired Student’s *t* test (**A**, **D**, **E**, **F**), and two-way ANOVA plus Bonferroni’s multiple comparisons test (**B**, **C**).

We then analyzed APAP-induced oxidative stress in liver tissues. ROS measurement by DHE staining in the sections of the liver tissue from mice treated APAP for 8 h showed that ROS level in Sirt6-Tg mice was much less than that in liver tissue from WT mice (Fig. 4A). These results indicated that Sirt6 gain-of-function could reduce the APAP-induced oxidative stress.

Activation of JNK is a currently acknowledged hallmark of APAP-induced ALF [26]. Reduced level of JNK phosphorylation after 2, 4, and 8 h of APAP administration in Sirt6-Tg mice correlated with reduced liver injury (Fig. 4B). The same results were observed in cultured primary hepatocytes in vitro, after treated with APAP (10 mM) for 8 h, phosphorylation of JNK was detected in WT hepatocytes, and the level of phosphorylated JNK in Sirt6 overexpressed hepatocytes was significantly decreased (Fig. 4C). Although necrosis is recognized as the major type of cell death, the apoptotic cell death still holds a place as an important contributor in APAP overdose-induced liver injury [27]. Further analysis revealed that cleaved caspase-3 and cleaved caspase-9, two effector molecules and well-verified markers for apoptosis [27], were apparently inhibited in the liver tissues from Sirt6-Tg mice liver compared with WT mice (Fig. 4D). Thus, our results suggested that Sirt6-Tg mice are protected from hepatocyte necrosis and apoptosis triggered by APAP.

### Sirt6 overexpression enhanced Nrf2/HO-1 signaling in APAP-induced liver injury

Nrf2/HO-1 signaling has been suggested as an important antioxidant pathway, which is essential for protecting against liver injury induced by APAP [28]. To further investigate the underlying mechanism of the Sirt6-mediated hepatoprotective effect, we assessed the regulatory of Sirt6 on the antioxidant Nrf2/HO-1 pathway. Our results indicated that Sirt6 overexpression not only significantly enhanced the Nrf2 protein expression but also induced an increase in the nuclear levels of Nrf2 in liver tissue (Fig. 4E, F). Furthermore, we found that Sirt6 overexpression could increase the HO-1 protein expression, which was a downstream target gene of Nrf2 (Fig. 4E). These data suggested that the antioxidant effect of Sirt6 overexpression in APAP-induced liver injury was likely mediated by activating the Nrf2/HO-1 pathway.

### Sirt6 overexpression inhibited Poly (ADP-ribose) polymerase 1 (PARP1) expression

**Fig. 5:**
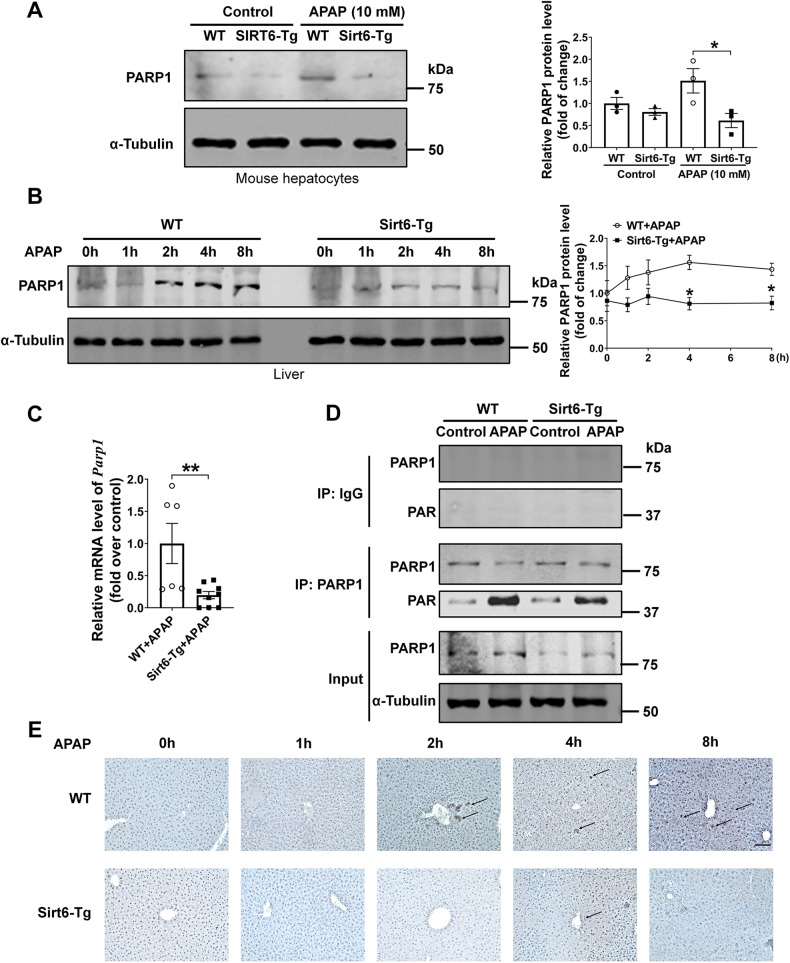
Sirt6 overexpression inhibited PARP1 expression. **A** Western blotting of PARP1 in WT and Sirt6-Tg mouse hepatocytes treated with APAP (10 mM) or PBS for 8 h. α-Tubulin was detected as a loading control. Results were presented as mean ± SEM (*n* = 3), ^*^P < 0.05. **B** Western blotting of PARP1 in the liver extracts from WT and Sirt6-Tg mice treated with APAP (500 mg/kg) or saline for the indicated times. α-Tubulin was detected as a loading control. Results were presented as mean ± SEM (*n* = 5 for each genotype at each time point), ^*^P < 0.05 compared with the WT group. **C** Relative hepatic mRNA expression of *Parp1* gene was measured by RT-qPCR assays in WT and Sirt6-Tg mice treated with APAP (500 mg/kg) for 8 h. Results were presented as mean ± SEM (*n* = 6–9), ^**^P < 0.01. **D** Representative Western blotting of auto-poly(ADP-ribosyl)ated PARP1 in the liver tissues from WT and Sirt6-Tg mice treated with APAP (500 mg/kg) for 8 h. **E** Immunohistochemicalanalysis of PARylated proteins in the liver tissues from WT and Sirt6-Tg mice treated with APAP (500 mg/kg) or saline for the indicated times. Statistical analysis was performed by two-way ANOVA plus Bonferroni’s multiple comparisons test (**A**, **B**), and unpaired Student’s *t* test (**C**).

PARP1 is a key regulator of multiple physiological and pathological processes. As a critical indicator of cell death, recent studies have revealed its vital role in APAP-induced liver injury [29]. As the most abundant isoform of PARP enzyme family, PARP1 leads the consumption of NAD^+^ and transfers the ADP-ribose moiety to acceptor proteins. Since Sirt6 is a NAD^+^-dependent histone deacetylase and mono-ADP ribosyltransferase enzyme [30, 31], it is conceivable to speculate the potential crosstalk between Sirt6 and PARP1 in APAP-induced liver injury. We first investigated the effect of Sirt6 overexpression on PARP1 expression. After treated with APAP (10 mM) for 8 h, PARP1 was greatly increased in mouse primary hepatocytes. Sirt6 overexpressed mouse primary hepatocyte showed a significant inhibition of PARP1 expression compared to that in WT hepatocytes (Fig. 5A). We next investigated the PARP1 level in mouse liver tissues. PARP1 expression was markedly increased in WT mouse livers after APAP administration for 2 h, while minimal PARP1 level was detected in Sirt6-Tg mouse livers compared with that in WT mice (Fig. 5B). RT-qPCR analysis also showed a trend of downregulation of *Parp1* gene expression in Sirt6 overexpression mouse livers under APAP administration for 8 h (Fig. 5C).

As a NAD^+^-dependent enzyme, PARP1 plays a vital role in cellular process through the Poly(ADP-ribosyl)ation (PARylation) of multiple proteins, and the first target protein of PARP1 PARylation is PARP1 itself [32]. We next checked the auto-PARylation of PARP1 in the liver tissues of APAP-treated mice. Co-immunoprecipitation assay results showed that the auto-PARylation level of PARP1 was markedly inhibited in the liver tissues of Sirt6-Tg mice compared with those of WT mice (Fig. 5D). Since the PARP1 activity is the marker of cell death, we next determined PARP1 activation by staining for PARylated proteins using immunohistochemistry, PAR staining intensity was significantly increased after APAP administration for 2 h in WT mice, while Sirt6-Tg mouse liver tissues showed a minimal PAR staining intensity (Fig. 5E). Taken together, these results indicated that Sirt6 overexpression not only decreases PARP1 expression but also inhibits PARP1-dependent PARylation during APAP-induced liver injury.

### Sirt6 overexpression ameliorated liver inflammation

**Fig. 6:**
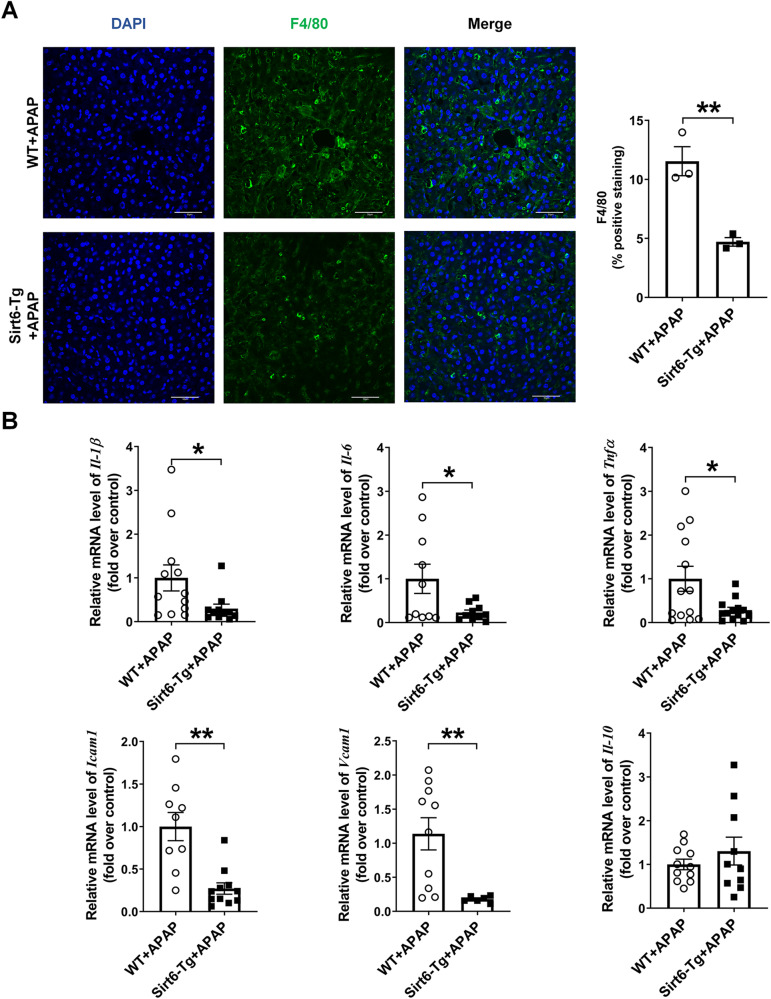
Sirt6 overexpression ameliorated liver inflammation. **A** Inflammation was assessed by F4/80 staining in the liver sections derived from WT and Sirt6-Tg mice treated with APAP (500 mg/kg) for 8 h (scale bar corresponds to 50 μm). Results were presented as mean ± SEM (*n* = 3), ^**^P < 0.01. **B** Relative hepatic mRNA expression of *Il-1β*, *Il-6*, *Tnfα*, *Icam1*, *Vcam1*, and *Il-10* genes were measured by RT-qPCR assays in the liver tissues from WT and Sirt6-Tg mice treated with APAP (500 mg/kg) for 8 h. Results were presented as mean ± SEM (*n* = 6–13), ^*^P < 0.05, ^**^P < 0.01. Statistical analysis was performed by unpaired Student’s *t* test.

The inflammation is also implicated in APAP-induced liver injury. So, we next analyzed the effect of Sirt6 overexpression on hepatic inflammation. We observed that liver inflammation was significantly reduced in Sirt6-Tg mice compared with that in WT mice as determined by the presence of F4/80 positive cells (macrophages) after 8 h of APAP administration (Fig. 6A). Likewise, the hepatic mRNA levels of the pro-inflammatory cytokines *Il-1β*, *Il-6*, *Tnfα*, *Icam1*, and *Vcam1* were also reduced in Sirt6-Tg mice after APAP administration for 8 h. By contrast, the mRNA levels of the anti-inflammatory markers *Il-10* was slightly increased in APAP-treated Sirt6-Tg mice compared with that in WT mice (Fig. 6B).

### Hepatic-specific Sirt6 overexpression in mice inhibited APAP-induced liver injury

**Fig. 7:**
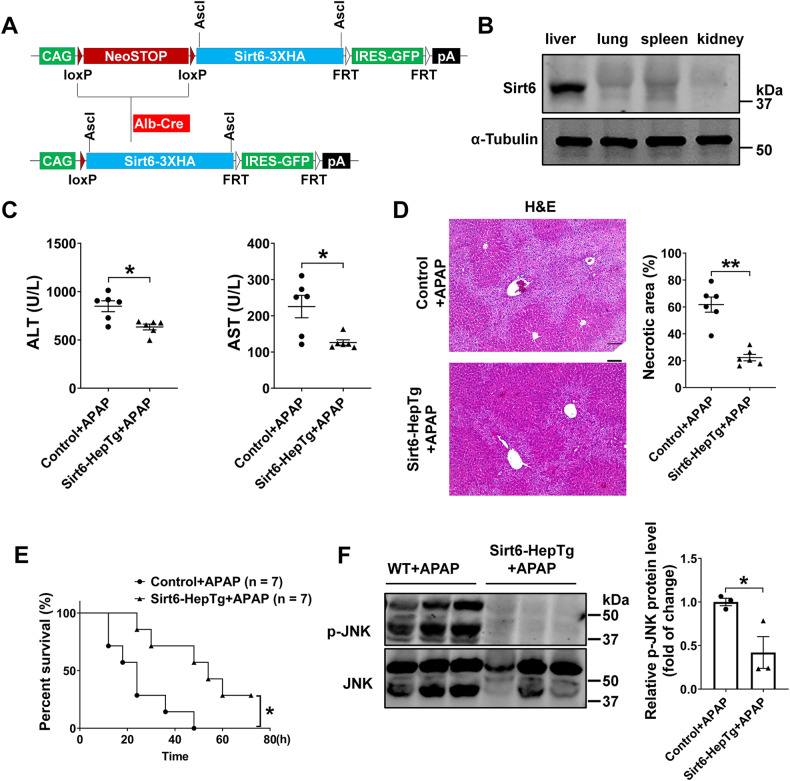
Overexpression of hepatic Sirt6 attenuated APAP-induced hepatotoxicity. **A** Schematic diagram for the generation of Sirt6-HepTg mice. **B** Western blotting analysis of Sirt6 in the liver, lung, spleen, and kidney from Sirt6-HepTg mice. α-Tubulin was detected as a loading control. **C** Serum ALT (left) and AST (right) activity in Control and Sirt6-HepTg mice treated with APAP (500 mg/kg) for 8 h. Each dot or triangle symbol represents a different mouse sample. Results were presented as mean ± SEM (*n* = 6), ^*^P < 0.05. **D** H&E staining of liver sections from Control and Sirt6-HepTg mice treated with APAP (500 mg/kg) for 8 h. 10×, scale bar corresponds to 200 µm. Quantification of necrotic areas by ImageJ software. Results were presented as mean ± SEM (n = 6), ^**^P < 0.01. **E** Survival rate of Control and Sirt6-HepTg mice treated with APAP (750 mg/kg) for 72 h (*n* = 7), ^*^P < 0.05. **F** JNK activation analyzed by Western blotting in the liver tissues from Control and Sirt6-HepTg mice treated with APAP (500 mg/kg) for 8 h. Blots were quantified and results were presented as mean ± SEM (*n* = 3), ^*^P < 0.05. Statistical analysis was performed by unpaired Student’s *t* test (**C**, **D**, **F**), and log-rank test (**E**).

To further substantiate the functional role of hepatic Sirt6 in APAP-induced liver injury, we generated hepatocyte-specific Sirt6 overexpression mouse model crossbreeding our conditional Sirt6-Tg mouse strain and Alb-Cre line (Sirt6-HepTg) (Fig. 7A). To confirm hepatic Sirt6 overexpression in Sirt6-HepTg mice, we performed Western blotting analysis of Sirt6 in multiple tissues including liver, lung, spleen, and kidney. Our data showed that the Sirt6 was efficiently overexpressed in the liver but not in other tissues (Fig. 7B). To investigate the role of hepatic Sirt6 in APAP-induced liver injury, Control mice and Sirt6-HepTg mice were both subjected to APAP administration for 8 h. Serum ALT and AST levels were significantly decreased in Sirt6-HepTg mice compared to those in Control mice (Fig. 7C). Histological analysis revealed that minimal necrotic areas in livers from Sirt6-HepTg mice relative to that in Control mice (Fig. 7D). Survival rate assay also showed that Sirt6-HepTg mice had an improved survival rate relative to Control mice within 72 h (Fig. 7E). Reduced levels of activated JNK after 8 h of APAP administration in Sirt6-HepTg mice correlated with reduced liver injury (Fig. 7F). These results were consistent with the phenotypes of global Sirt6-Tg mice and demonstrated the specific role of hepatic Sirt6 in the protection against the APAP-induced liver injury.

### Pharmacological Sirt6 activator prevented and reversed APAP-induced liver injury

**Fig. 8:**
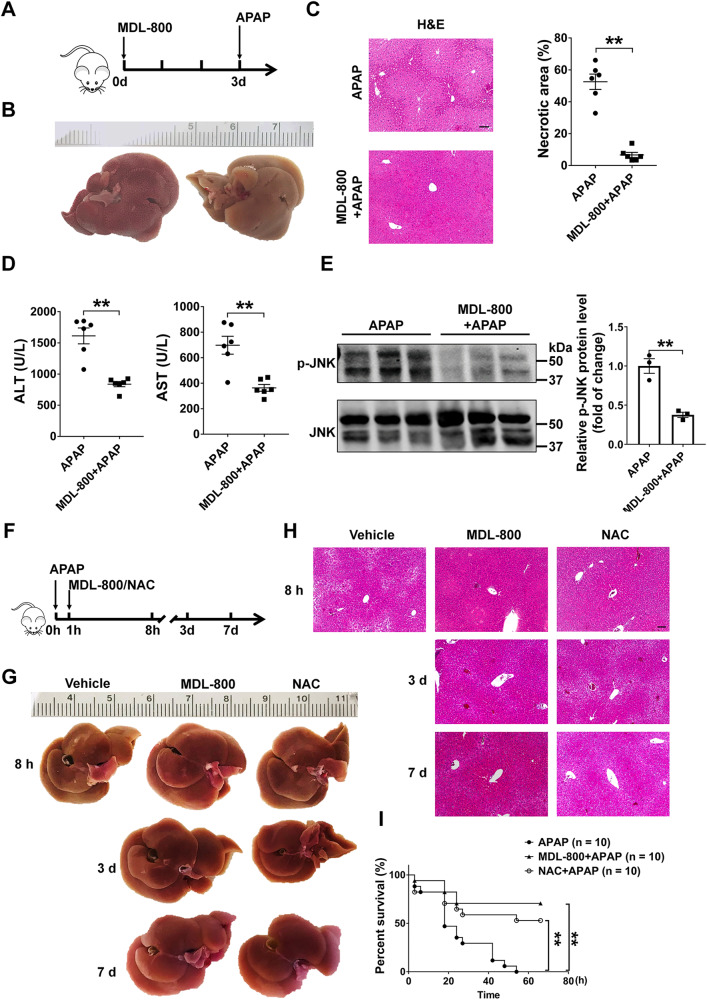
The treatment of the Sirt6 activator prevented and treated APAP-induced hepatotoxicity. **A**–**E** normal C57BL/6J mice were subjected to 3 days (once a day) pretreatment with vehicle or MDL-800 (100 mg/kg) and followed with the treatment with APAP (500 mg/kg) for 8 h. **A** Schematic of the experimental design. **B** Representative images showed the appearance of mouse livers. **C** H&E staining of liver sections from mice treated with vehicle or MDL-800. 10×, scale bar corresponds to 200 µm. Quantification of necrotic areas by ImageJ software. Results were presented as mean ± SEM (*n* = 6), ^**^P < 0.01. **D** Serum ALT (left) and AST (right) activity in vehicle and MDL-800 treated mice. Each dot or triangle symbol represents a different mouse sample. Results were presented as mean ± SEM (*n* = 6), ^**^P < 0.01. **E** JNK activation analyzed by Western blotting in WT mice treated with vehicle or MDL-800 (100 mg/kg). Results were presented as mean ± SEM (*n* = 3), ^**^P < 0.01. **F**–**I** Normal C57BL/6J mice were treated with APAP (500 mg/kg) for 1 h then subjected intraperitoneal injected with MDL-800 (100 mg/kg), NAC (100 mg/kg) or vehicle separately and followed for 7 days to score the liver injury. **F** Schematic of the experimental design. **G** Representative images showed the appearance of mouse livers. **H** H&E staining of liver sections from mice treated with MDL-800 (100 mg/kg), NAC (100 mg/kg), or vehicle. 10×, scale bar corresponds to 200 µm. **I** Survival rate of mice. Normal C57BL/6J mice were treated with APAP (750 mg/kg) for 1 h, then subjected intraperitoneal injected with MDL-800 (100 mg/kg), NAC (100 mg/kg), or vehicle separately and followed for 66 h to score the survival rate (*n* = 10), ^**^P < 0.01. Statistical analysis was performed by unpaired Student’s *t* test (**C**, **D**, **E**), and log-rank test (**I**).

Recently study has identified that MDL-800 is a selective small-molecule Sirt6 activator [33]. To investigate whether pharmacological activation of Sirt6 could ameliorate APAP-induced liver injury, we assessed the effect of MDL-800 in vivo. WT mice were once-daily intraperitoneal injected with MDL-800 (100 mg/kg) for 3 days before 8 h APAP administration. Histological analysis showed minimal APAP-induced necrotic areas in livers from mice treated with MDL-800 relative to vehicle treatment (Fig. 8A–C). Serum transaminases analysis also revealed that the treatment with MDL-800 decreased ALT and AST levels (Fig. 8D). Moreover, MDL-800 treatment inhibited JNK activation in the liver tissues (Fig. 8E). These results suggested that Sirt6 activator MDL-800 could prevent APAP-induced liver damage.

Since the NAC is the only antidote approved for APAP overdose and it reduces mortality [34], we compared the efficiency of MDL-800 and NAC on APAP overdose-induced liver injury. C57BL/6J mice were treated with APAP (500 mg/kg) by intraperitoneal injection, after 1 h, mice were intraperitoneal injected with MDL-800 (100 mg/kg), NAC (100 mg/kg), or vehicle respectively (Fig. 8F) and followed for 7 days to score the liver injury. Both MDL-800 and NAC could protect mice from liver injury after APAP treatment at 8 h (Fig. 8G, H). Mice treated with vehicle were all died 3 days after APAP administration. We observed and compared the effect of MDL-800 and NAC on the mice that lived to the third days and the seventh days after APAP administration. The results indicated that MDL-800 had a better protective effect on liver injury than NAC (Fig. 8G, H). In addition, both MDL-800 and NAC significantly improve survival rate of mice challenged with APAP, but MDL-800 seems to work better (Fig. 8I). Together, these results highlight the beneficial effects of MDL-800 on APAP-induced liver injury, suggesting that MDL-800 could be a promising therapeutic agent for ALF.

### Sirt6 overexpression ameliorated BDL-induced liver injury

BDL is a commonly used surgical model of ALF [35]. To confirm the protection of Sirt6 on liver failure, we then examined the role of hepatic Sirt6 on BDL-induced liver injury, we performed BDL in both Control mice and Sirt6-HepTg mice that were sacrificed at 1, 3, and 7 days after surgery. Histopathological analysis confirmed the liver damage by BDL as evidenced by wide areas of necrosis in the livers of Control mice at 1, 3, and 7 days after surgery. We observed that much less centrilobular necrotic lesions in Sirt6-HepTg mice compared with those of Control mice (Fig. S2). These results showed that hepatic Sirt6 overexpression could significantly ameliorate BDL-induced liver damage.

### Hepatic-specific Sirt6 knockout in mice exacerbated APAP-induced and BDL-induced liver injury

To further investigate the crucial role of endogenous Sirt6 in liver injury, we used the loss-of-function approach by generating a hepatic-specific Sirt6 knockout (Sirt6-HepKO) mouse model using a floxed Sirt6 mouse strain and an Alb-Cre line. Sirt6-Flox and Sirt6-HepKO mice were subjected to either APAP (500 mg/kg) treatment for 8 h or BDL for 1 day. Histological results indicated that Sirt6-HepKO mice showed more serious necrosis in the liver (Fig. S3), confirming the essential role of hepatic Sirt6 in the protection against the ALF.

## Discussion

In this work, we revealed a crucial role of Sirt6 in protecting against both APAP-induced and BDL-induced ALF. Our investigation has begun from an interesting observation that hepatic Sirt6 expression significantly downregulated in the livers of human and mice with acute liver failure. The rescue and maintenance of Sirt6 expression in the Sirt6-Tg mice after APAP administration protected against the pathological changes during APAP-induced liver toxicity by limiting oxidative stress and inflammation. The protective effect of hepatic Sirt6 was further validated in Sirt6-HepTg mice and in the mice treated with the specific Sirt6 activator MDL-800. Hepatic-specific Sirt6 knockout in mice exacerbated, but hepatic overexpression of Sirt6 ameliorated, acute liver damages-induced by both APAP and BDL. Furthermore, the Sirt6 activator MDL-800 presented better therapeutic potential to alleviate liver injury in mice than NAC, which is the only proved antidote for APAP overdose up to now. Taken together, these results demonstrate that Sirt6 could be an important therapeutic target for the treatment of ALF.

Hepatic apoptosis and necrosis have been critically implicated in APAP-induced ALF. Once ingested, APAP is metabolite in hepatocytes and eliminated by glucuronidation and sulfation into urine. The main enzymes involved in APAP metabolism were CYP1A2, CYP2E1, and CYP3A11. NAPQI, the electrophilic metabolite of APAP, trigger the depletion of GSH and generation of APAP-adducts, which eventually result in liver damage. Though the exact mechanisms of APAP-induced liver injury remain elusive, apoptosis and necrosis are recognized as two major subroutines of cell death triggered by APAP overdose [27]. In this study, we found that humanized transgenic Sirt6 overexpression had no effect on the levels of liver NAPQI nor cytochromes P450s, but markedly inhibited necrosis and apoptosis of hepatocytes in APAP-treated mice and primary hepatocytes. The protection of Sirt6 overexpression on APAP-induced hepatocyte death, which results from downregulation of both necrosis and apoptosis, inclines us to believe that Sirt6 has a potent protective effect on various types of cell death during APAP overdose.

Several death-associated signal pathways have been proposed to be responsible for APAP-induced hepatoxicity [36, 37]. Oxidative stress is speculated as a crucial procedure upon APAP overdose. Accumulation of NAPQI generated ROS in liver upon APAP administration. The combined reaction of GSH depletion and oxidative stress could inhibit Nrf2/HO-1 signaling pathway and activate the stress-activated protein kinase JNK, which both were reported to amplify APAP-induced hepatotoxicity [38]. Activated JNK regulates multiple cellular responses through binding with c-JUN and enhancing its transcriptional activity. In this study, we found that Sirt6 overexpression not only inhibited oxidative stress by enhancing the Nrf2/HO-1 pathway, but also suppressed JNK activation in the liver tissues, which both contributed to the protection of Sirt6 overexpression in APAP-induced ALF.

Since JNK has been reported to participate in inflammation, and inflammation is an essential part of the pathogenesis of APAP-induced liver damage [39]. Accumulation of NAPQI and subsequent ROS generation both stimulate inflammatory cell infiltration and trigger an increase of inflammatory mediators in the livers, which leads to the amplification of liver damage. As Sirt6 is an anti-inflammatory regulator, we further tested the effect of Sirt6 overexpression on inflammation. Results indicated that Sirt6 significantly reduced the infiltration of macrophages in liver and suppressed the mRNA levels of pro-inflammatory cytokines in the liver tissues. Our finding was consistent with recent reports indicating that Sirt6 protects against tissue injury upon different stimulation [22].

PARP1 is another stress response regulator during multiple stress processes. Although PARP1 has DNA repair effect upon mild stimulation, over activation of PARP1 directly contributes to cell death. As a nuclear enzyme, PARP1 has been identified to trigger necrosis through multiple mechanisms, such as depleting NAD^+^ supply of cell and promoting JNK activation [40]. PARP1 is the main member of PARP family, which catalyzes the formation of various poly(ADP-ribosylated) proteins, concomitant consumption of NAD and necrosis, and has been shown to regulate oxidative stress and cell death in liver [41]. NAPQI is known to trigger NAD^+^ hydrolysis to ADP-ribose, and the PARP1 inhibitor suppressed APAP-induced hepatotoxicity [29]. In our study, we demonstrated that Sirt6 markedly inhibited the protein and mRNA level of *Parp1* in the liver tissues from APAP-treated mice. Furthermore, we found that Sirt6 overexpression also suppressed the PARP1-mediated poly(ADPribosyl)ation. Taken together, our results suggest that a decrease in PARP1 expression and PARP1-dependent PARylation is most likely a contributing factor in Sirt6’s ability to prevent APAP-induced liver damage.

BDL could induce acute cholestatic liver injury, and its underlying mechanism is different from APAP-induced liver injury. Our results show that hepatic Sirt6 also has a significant protective effect against BDL-induced liver damage. A previous study has shown that Sirt6 plays a critical protective role against alcohol-induced liver injury [22]. Since all three mouse models share similar phenotypes, which are characterized by hepatic inflammation, oxidative stress, and necrosis, it is conceivable to speculate that Sirt6 might have protective action on various conditions of liver failure.

In conclusion, our results revealed a crucial role of hepatic Sirt6 in the protection of acute hepatotoxicity. Genetic overexpression of Sirt6 and the pharmacological activation of Sirt6 ameliorated oxidative stress, inflammation, and liver injury. The potential mechanisms whereby Sirt6 protects against hepatotoxicity include that Sirt6 inhibits JNK and caspase 3/9 activation, enhanced Nrf2/HO-1 pathway and attenuates PARP1 expression and activity. Therefore, Sirt6 orchestrates the safeguard against hepatotoxicity and may serve as a new therapeutic target for the ALF.

## Materials and methods

### Human hepatic tissue samples

Human tissues were obtained under informed consent using protocols approved by the Institutional Review Board of 2nd Xiangya Hospital of Central South University. Human liver samples were obtained from healthy individuals and patients who undergo a liver transplant with liver failure from the Department of Pathology, the 2nd Xiangya Hospital of Central South University (Table S1). Informed written consent was obtained from each patient.

### Animal treatment

All animal experiments were approved by the Institutional Animal Care and Use Committee of the University of Rochester Medical Center.

The Sirt6 transgene was constructed in a specially designated CTV vector for gain-of-function at Rosa26 locus (Addgene, Cambridge, MA). The final construct consisted of the strong universal promoter designated CAG upstream of a transcriptional/translational stop cassette (*neostop*) flanked by loxP DNA cis-elements and followed by human Sirt6-3HA cDNA (Fig. 2A). This transgenic mouse line was generated in Lin Gan’s laboratory by the Mouse Genome Editing Resource offers services of University of Rochester. Mice expressing Cre recombinase under control of the EIIa-Cre promoter/enhancer were purchased from the Jackson Laboratory (Bar Harbor, ME, USA). To generate global Sirt6 transgenic mice (Sirt6-Tg), mice containing the Sirt6-3HA transgene were bred with C57BL/6J EIIa-Cre mice (Fig. 2A), we successfully bred and divided littermate control mice into groups consisting of wild type (WT), Sirt6-Tg mice according to genotype identification analysis.

To generate hepatic-specific Sirt6 transgenic mice (Sirt6-HepTg), mice containing the Sirt6-3HA transgene were bred with mice expressing Cre recombinase under control of the Alb-Cre promoter/enhancer (Jackson Laboratory, Bar Harbor, ME) (Fig. 7A), After weaning, littermate control mice (referred to as Control) and Sirt6-HepTg mice were used for further experiments.

Sirt6-Flox (Sirt6^flox/flox^) mice were purchased from the Jackson Laboratory. LoxP recombination sites flank exons 2 and 3 of the Sirt6 gene [42]. To generate liver-specific Sirt6 knockout mice (Alb-Cre/Sirt6^flox/flox^, defined as Sirt6-HepKO), Sirt6^flox/flox^ mice (defined as Sirt6-Flox) were bred with mice expressing Cre recombinase under control of the Alb-Cre promoter/enhancer (Jackson Laboratory), SIRT6^flox/flox^ littermates were used as the control.

Mice were fasting overnight before being injected with APAP. APAP (Sigma-Aldrich, St. Louis, MO) was dissolved in saline at 55 °C. Mice were intraperitoneally injected with APAP (500 mg/kg for protection experiments or 750 mg/kg for lethality test) or saline. Serum samples and liver tissue of mice were collected at 0, 1, 2, 4, 8 h after APAP administration.

Sirt6 activator MDL-800 (Aobious, Gloucester, MA) was dissolved in vehicle (5% DMSO, 30% PEG-400, or 65% saline, pH 7–8) and was once-daily intraperitoneal injection. N-acetylcysteine (NAC, Sigma-Aldrich) was dissolved in saline and was once-daily intraperitoneal injection.

Sirt6^flox/flox^ (Sirt6-Flox) and Sirt6-HepKO mice (at least 12 weeks of age) were subjected to BDL without cholecystectomy [43] for up to 7 days.

### Hematoxylin and eosin (H&E) staining

Mice livers were fixed in 4% paraformaldehyde and embedded in paraffin (Sigma-Aldrich), sectioned livers (10 μm) were prepared and used for H&E staining. Quantification of necrotic areas were calculated using Image J software (http://imagej.nih.gov).

### Analysis of serum alanine aminotransferase (ALT) and aspartate aminotransferase (AST) activity

Serum samples were diluted, and ALT and AST activity were measured using commercial ALT kit and AST kit (Sigma-Aldrich).

### NAPQI detection

Hepatic NAPQI was determined using an ELISA kit from RYBIO, following the manufacturer’s instructions.

### Analysis of liver glutathione (GSH) level

GSH assay kit was purchased from Enzo Life Sciences. The data were expressed as micromoles per milligram of protein.

### Terminal deoxynucleotidyl transferase dUTP nick-end labeling (TUNEL) analysis

TUNEL assay was performed in frozen liver sections and primary hepatocytes using in situ TUNEL assay kit (Invitrogen, Carlsbad, CA), according to the manufacturer’s instructions. Images were acquired using an Olympus BX81 system microscope.

### Reactive oxygen species (ROS) determination

Liver tissues were harvested and embedded in Tissue-Tek optimal cutting temperature compound (O.C.T, Sakura Finetek, Torrance, CA). Cryosections of unfixed frozen segments were obtained and incubated with dihydroethidium (DHE, 10 μM) (Sigma-Aldrich) at 37 °C for 30 min. DAPI (4′,6-diamidino-2-phenylindole, dihydrochloride; Vector Laboratories, Burlingame, CA) was used for nuclear counterstaining. Images were acquired using an Olympus BX81 system microscope.

### Western blotting assay

Protein lysates from isolated liver tissues and hepatocytes homogenates were prepared using lysis buffer containing 10 mM Tris-HCl, 5 mM EDTA, 50 mM NaCl, 30 mM disodium pyrophosphate, 50 mM NaF, 100 μM Na_3_VO_4_, 1% Triton X-100, 1 mM phenylmethylsulfonyl fluoride, 10 μg/mL leupeptin, and 10 μg/mL aprotinin pH 7.6. After centrifugation at 12,000 *g* for 10 min. Supernatant was collected and separated by SDS-PAGE and transferred onto PVDF membranes (Millipore, Burlington, MA, USA). Membranes were blocked in 5% bovine serum albumin (BSA, Sigma-Aldrich) in TBST (Tris-buffered saline, 0.1% Tween 20) for 1 h at room temperature and incubated with different primary antibodies overnight at 4 °C [44].

Specification of the source, dilution, and application of both primary and secondary antibodies are listed in Table S2. Membranes were washed three times and incubated with IRDye® 680RD Goat anti-Mouse IgG (H + L) or IRDye® 800CW Goat anti-Rabbit IgG (H + L) (1:10,000 dilution in 1xTBST; LI-COR) at room temperature for 30 min. Images were visualized by using an Odyssey Infrared Imaging System (LI-COR).

### Immunostaining analysis

Liver tissues were fixed and paraffin embedding. Cryosections were prepared and used for immunofluorescence and immunohistochemistry.

Nonspecific antibody binding was blocked by goat serum. High-mobility group box 1 (HMGB1) and F4/80 staining was performed with anti-HMGB1 and anti-F4/80 antibody (Cell Signaling Technology, Danvers, MA) at 1:100 dilution in a humidified chamber at 4 °C overnight. After incubation with Alexa-conjugated secondary antibodies (Invitrogen) at 1:1000 dilution for 1 h at room temperature, signals were visualized by confocal microscopy, and DAPI was used for nuclear counterstaining. Quantification was using Image J software.

Human liver Sirt6 staining was performed using anti-Sirt6 antibody (Cell Signaling Technology) and immunohistochemistry kit (Abcam, Cambridge, UK) according to the manufacturer’s instructions. Images were created using CaseViewer software (3D Histech, Budapest, Hungary).

Mice liver PAR staining was performed using anti-PAR antibody (Tulips Biolabs, Lansdale, PA) and immunohistochemistry kit (Abcam) according to the manufacturer’s instructions. Quantification was using Image J software.

### Primary hepatocyte isolation and culture

Mouse primary hepatocytes were isolated as described [45]. Hepatocytes were cultured in DMEM supplemented with 10% FBS (Thermo Fisher Scientific, Waltham, MA), 100 U/mL penicillin, 100 μg/mL streptomycin, and were maintained at 37 °C in 5% CO_2_. Hepatocytes were treated in 6-well plates with APAP (10 mM) diluted in saline for 8 h. Saline alone was used as control.

### Co-immunoprecipitation

Mice liver protein lysates were incubated with the indicated antibodies against PARP1 or unspecific IgG at 4 °C overnight. Mix the slurry well and add 70–100 μL of the protein A/G agarose (Thermo Fisher Scientific) to each sample for another 4 h at 4 °C. Centrifuge and wash the immunoprecipitants five times and the pellets were suspended in SDS loading buffer and analysis by western blotting.

### Real-time quantitative PCR (RT-qPCR)

Total RNA was extracted from the liver tissues of each mice liver using Trizol Lysis Reagent (Invitrogen) according to the manufacturer’s instruction. Complementary DNA was prepared using a High-Capacity cDNA Reverse Transcription Kit (Thermo Fisher Scientific). RT-qPCR was performed on CFX96 Real-Time PCR detection system (Bio-Rad, Hercules, CA) with SYBR Green (Thermo Fisher, Waltham) and all the primers (Table S3). The comparative cycle threshold (Ct) method (2^−ΔΔCt^) was used to determine the relative mRNA expression of target genes after normalization to housekeeping gene *Gapdh* or *β-actin* [46].

### Statistical analysis

Data was analyzed using GraphPad Prism 5 software (GraphPad Software, Inc) and expressed as mean ± SEM. Differences between the two groups were analyzed by two-tailed Student’s *t* test. Differences between multiple group comparisons for single and two variables were analyzed by One-way ANOVA plus Dunnett’s test or two-way ANOVA plus Bonferroni test, respectively. A log-rank test was used to determine the significance of mouse survival. *P* values less than 0.05 (*P* < 0.05) was considered statistically significant.

### Supplementary information

Supplemental information

Original Data File

aj-checklist

## Data Availability

All data are available within the article, Supplementary Information or Original Data file.
