# Supplementary material for: Hepatic Sirt6 activation abrogates acute liver failure
Source: Cell Death Dis. 2024 Apr 22;15(4):283. doi: 10.1038/s41419-024-06537-5 (PMC11035560; doi:10.1038/s41419-024-06537-5)
Supplement: Supplementary file 1 — Supplemental information [file 41419_2024_6537_MOESM1_ESM.docx]

**Supplementary Information**

**
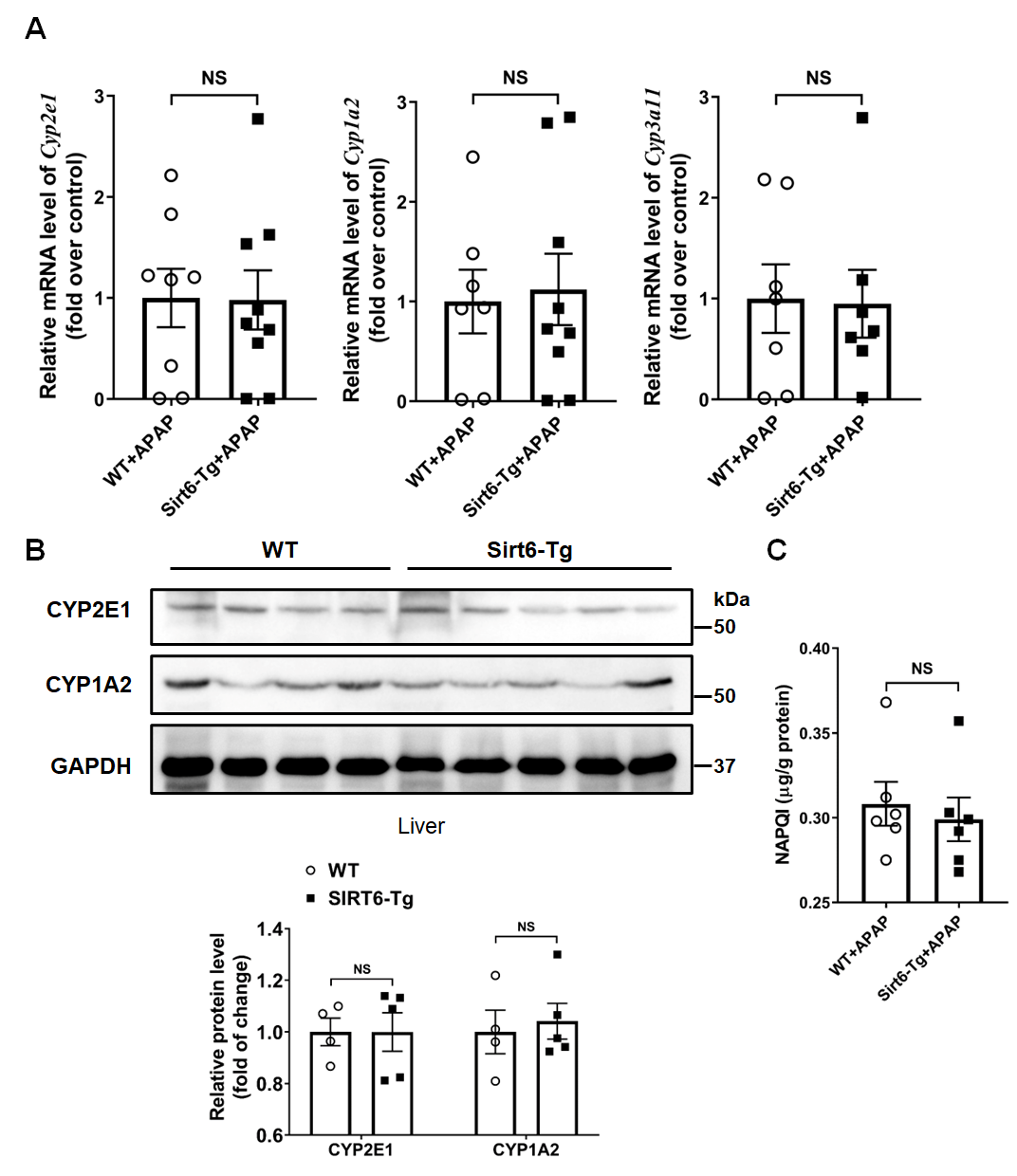
**

**Fig. S1 Effects of Sirt6 overexpression on hepatic expressions of CYP2E1, CYP1A2, and CYP3A11 in APAP-treated mice.** **A** Relative hepatic mRNA expression of *Cyp2e1*, *Cyp1a2*, and *Cyp3a11* genes were measured by RT-qPCR assays in WT and Sirt6-Tg mice treated with APAP (500 mg/kg) for 8 hours. Results were presented as mean ± SEM (n = 7-9 for each group). **B** Western blotting of CYP2E1 and CYP1A2 in the liver tissues from WT and Sirt6-Tg mice treated with APAP (500 mg/kg) for 8 hours. Blots were quantified and results were presented as mean ± SEM (n = 4-5). **C** ELISA assay of NAPQI levels in the liver tissues from WT and Sirt6-Tg mice treated with APAP (500 mg/kg) for 8 hours. Statistical analysis was performed by unpaired Student’s t-test. NS, no significance.

**
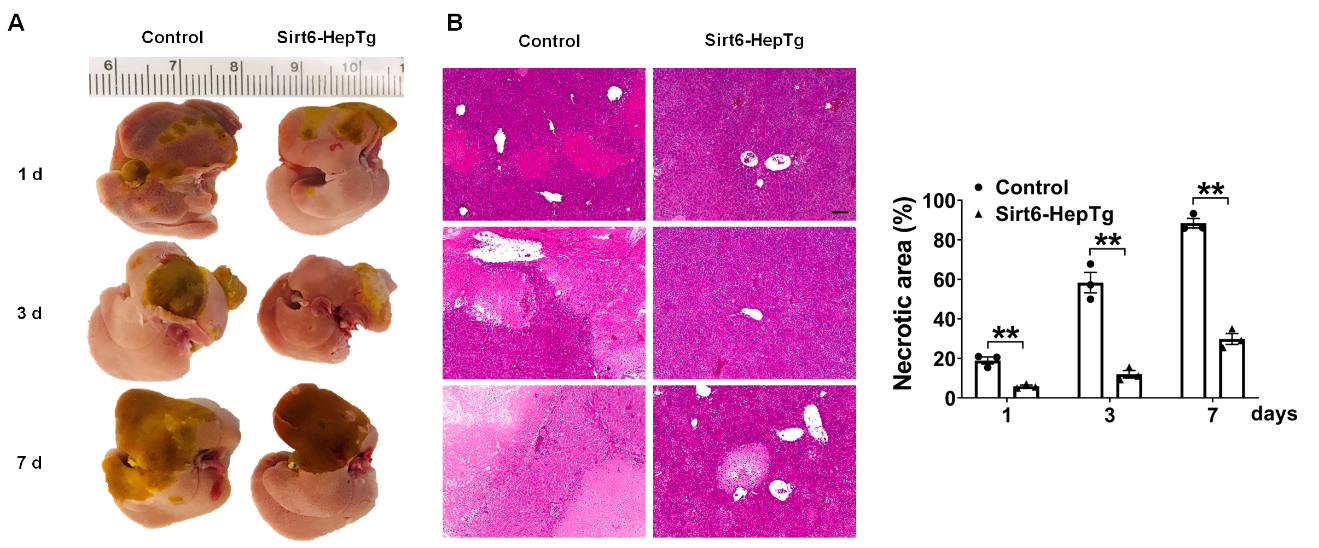
**

**Fig. S2 Overexpression of hepatic Sirt6 attenuated** **liver injury in BDL mice.** Control and Sirt6-HepTg mice were subjected to BDL for 7 days. (A) photographs of representative mouse liver were obtained 1, 3, 7 days after BDL. (B) Liver necrosis was assessed by H&E staining. Scale bar: 200 μm. The area of necrosis was measured (n = 3), ^**^*P* < 0.01. Statistical analysis was performed by unpaired Student’s t-test.

**
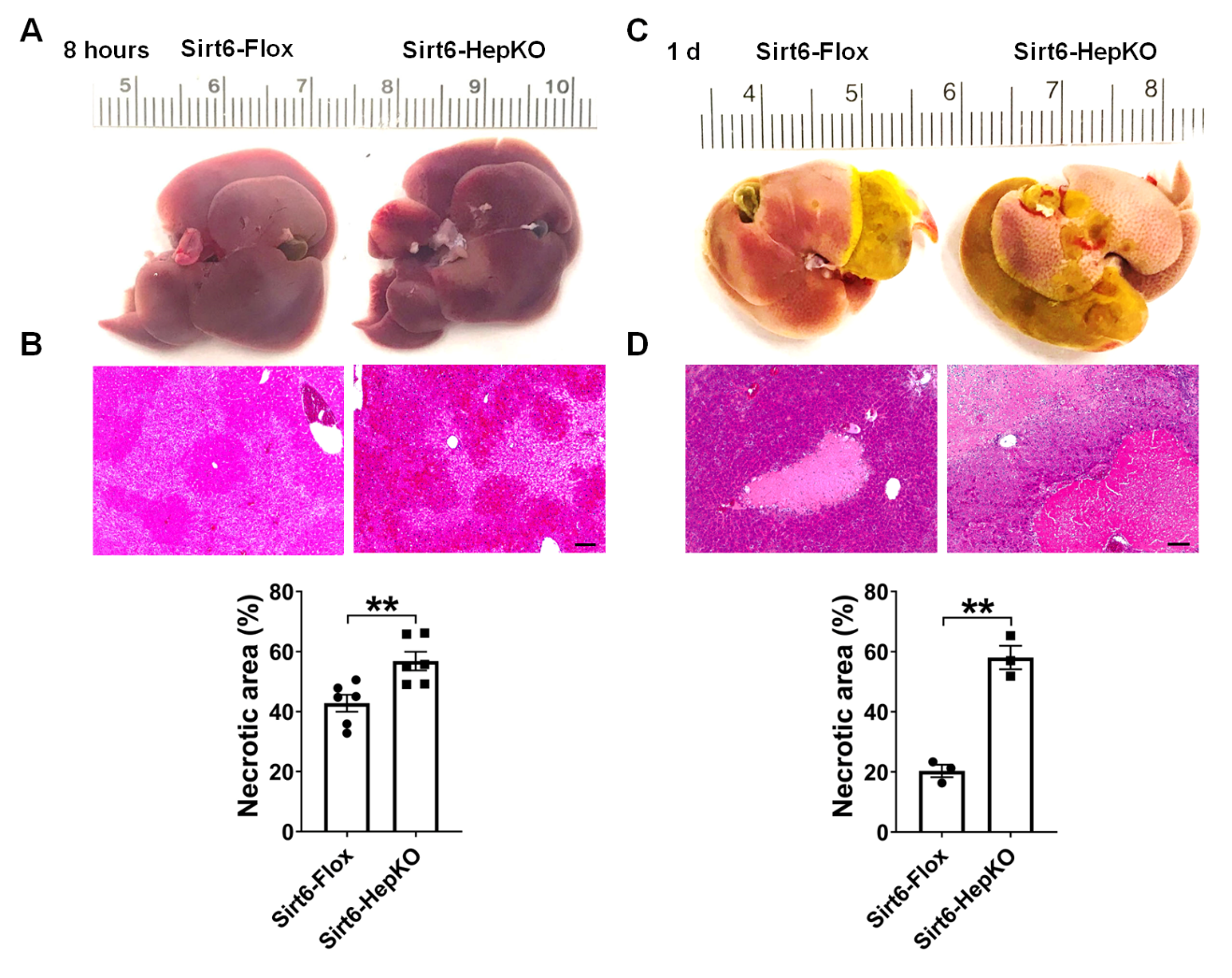
**

**Fig. S3 Hepatic Sirt6 deficiency aggravate APAP-induced and BDL-induced liver injury.** Sirt6-Flox and hepatic Sirt6 knockout (Sirt6-HepKO) mice were injected intravenously with APAP (500 mg/kg) for 8 hours. (A) Representative images showed the appearance of mouse livers. (B) Liver necrosis was assessed by H&E staining.

10x, scale bar corresponds to 200 µm. Quantification of necrotic areas by ImageJ software. Results were presented as mean ± SEM (n = 6), ***P* < 0.01.

WT and hepatic Sirt6 knockout (Sirt6-HepKO) mice were BDL for 1 day. (C) Representative images showed the appearance of mouse livers. (D) Liver necrosis was assessed by H&E staining. 10x, scale bar corresponds to 200 µm. Quantification of necrotic areas by ImageJ software. Results were presented as mean ± SEM (n = 3), ^**^*P* < 0.01. Statistical analysis was performed by unpaired Student’s t-test.

**Table S1 Characteristics of the liver failure patients.**

| Patients | Aetiology | Age | Sex | ALT | AST | ALB | TBIL | WBC | PLT |
| --- | --- | --- | --- | --- | --- | --- | --- | --- | --- |
| ID |  | (yr) |  | (U/L) | (U/L) | (g/L) | (μmol/L) | (X10^9^/L) | (X10^9^/L) |
| 1725487 | Hepatitis E | 42 | M | 73.8 | 79.9 | 35.8 | 76.2 | 1.77 | 26 |
| 1700082 | Acute toxic hepatitis | 48 | M | 35.4 | 172.5 | 30.7 | 37.2 | 1.52 | 33 |
| 1723234 | Drug-induced liver injury | 51 | M | 59.6 | 152.4 | 37.4 | 96.8 | 5.7 | 34 |
| 1727125 | Drug-induced liver injury | 46 | F | 42.8 | 123.9 | 28.3 | 77.7 | 1.95 | 22 |
| 1773743 | Drug-induced liver injury | 45 | F | 57.4 | 97.4 | 24.9 | 133.9 | 6.49 | 29 |

ALT, alanine transaminase; AST, aspartate transaminase; ALB, albumin; TBIL, total bilirubin; WBC, white blood cells; PLT, platelet.

**Table S2 Source of primary and secondary antibodies used for Western blotting.**

| Antibodies | Supplier, Cat. No., host | Dilution |
| --- | --- | --- |
| Sirt6 | CST, #12486, rabbit | 1:1, 000 (in 5%BSA) |
| JNK | CST, #9252, rabbit | 1:1, 000 (in 5%BSA) |
| p-JNK | CST, #9255, mouse | 1:1, 000 (in 5%BSA) |
| PARP1 | Trevigen, #4338-MC-50, mouse | 1:1, 000 (in 5%BSA) |
| Cleaved caspase-3 | CST, #9661, rabbit | 1:1, 000 (in 5%BSA) |
| Cleaved caspase-9 | CST, # 20750, rabbit | 1:1, 000 (in 5%BSA) |
| CYP2E1 | Abcam, ab28146 | 1:1, 000 (in 5%BSA) |
| CYP1A2 | CST, #14719, mouse | 1:1, 000 (in 5%BSA) |
| α-Tubulin | Sigma, #T5168 | 1:10, 000 (in 5%BSA) |
| PAR | ProteinTech, 16124-1-AP | 1:1, 000 (in 5%BSA) |
| Nrf2 | NOVUS, NBP1-32822 | 1:1, 000 (in 5%BSA) |
| HO-1 | Abcam, ab13243 | 1:1, 000 (in 5%BSA) |
| Lamin B1 | Santa Cruz, sc-374015 | 1:1, 000 (in 5%BSA) |
| IRDye® 680RD Goat anti-Mouse IgG (H + L), 0.5 mg | Licor, #926-68070, goat | 1:10, 000 (in 1XTBST) |
| IRDye® 800CW Goat anti-Rabbit IgG (H + L), 0.5 mg | Licor, #926-32211, goat | 1: 10, 000 (in 1XTBST) |
| IRDye® 680RD Donkey anti-Goat IgG (H + L), 0.5 mg | Licor, #926-68074, donkey | 1: 10, 000 (in 1XTBST) |
| IRDye® 800CW Donkey anti-Rabbit IgG (H + L), 0.5 mg | Licor, #926-32213, donkey | 1: 10, 000 (in 1XTBST) |
| IRDye® 680RD Donkey anti-Chicken IgG (H + L), 0.5 mg | Licor, #926-68075, donkey | 1: 10, 000 (in 1XTBST) |

**Table S3 Primers sequences.**

| Primer for | Primer Sequence 5’ to 3’ |
| --- | --- |
| *Parp1* | Forward: GGAAAGGGATCTACTTTGCCG |
|  | Reverse: TCGGGTCTCCCTGAGATGTG |
| *Cyp1a2* | Forward: AGTACATCTCCTTAGCCCCAG |
|  | Reverse: GGGTCCGGGTGGATTCTTC |
| *Cyp2e1* | Forward: CGTTGCCTTGCTTGTCTGGA |
|  | Reverse: AAGAAAGGAATTGGGAAAGGTCC |
| *Cyp3a11* | Forward: GACAAACAAGCAGGGATGGAC |
|  | Reverse: CCAAGCTGATTGCTAGGAGCA |
| *Il-1β* | Forward: AACCTGCTGGTGTGTGACGTTC |
|  | Reverse: CAGCACGAGGCTTTTTTGTTGT |
| *Il-6* | Forward: ACAACCACGGCCTTCCCTACTT |
|  | Reverse: CACGATTTCCCAGAGAACATGTG |
| *Tnf-α* | Forward: TACTGAACTTCGGGGTGATTGGTCC |
|  | Reverse: CAGCCTTGTCCCTTGAAGAGAACC |
| *Vcam-1* | Forward: GCACTCTACTGCGCATCTT |
|  | Reverse: CACCAGACTGTACGATCCTTTC |
| *Icam-1* | Forward: GGGAATGTCACCAGGAATGT |
|  | Reverse: CTGATCTTTCTCTGGCGGTTAT |
| *Il-10* | Forward: ACAGCCGGGAAGACAATAAC |
|  | Reverse: CAGCTGGTCCTTTGTTTGAAAG |
| *Gapdh* | Forward: AACAGCAACTCCCACTCTTC |
|  | Reverse: CCTGTTGCTGTAGCCGTATT |
| *β-actin* | Forward: GTGACGTTGACATCCGTAAAGA |
|  | Reverse: GCCGGACTCATCGTACTCC |
