# Supplementary material for: Hepatic Sirt6 activation abrogates acute liver failure
Source: Cell Death Dis. 2024 Apr 22;15(4):283. doi: 10.1038/s41419-024-06537-5 (PMC11035560; doi:10.1038/s41419-024-06537-5)

**Figure 1B**


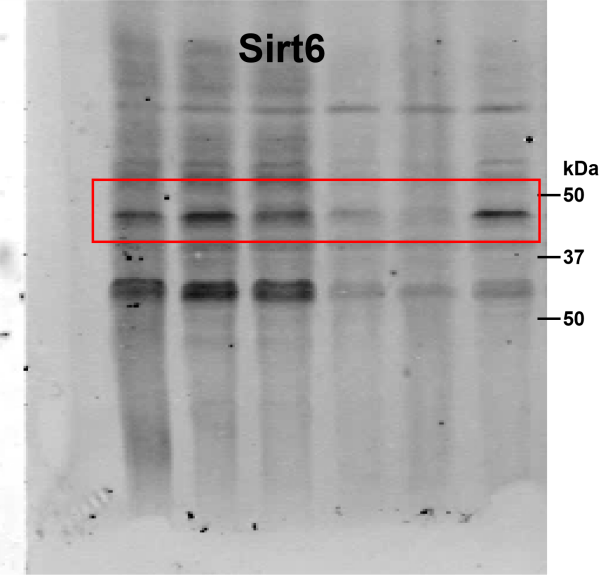

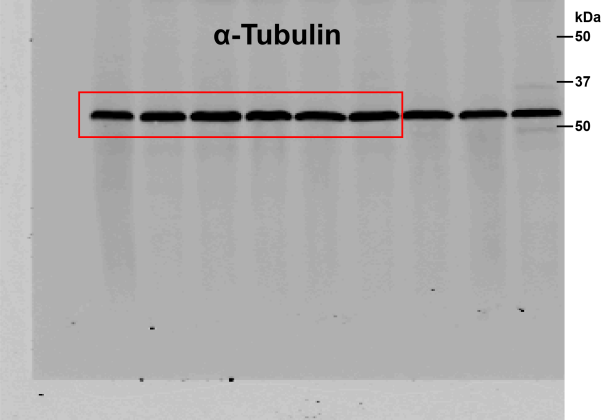


**Figure 1C**


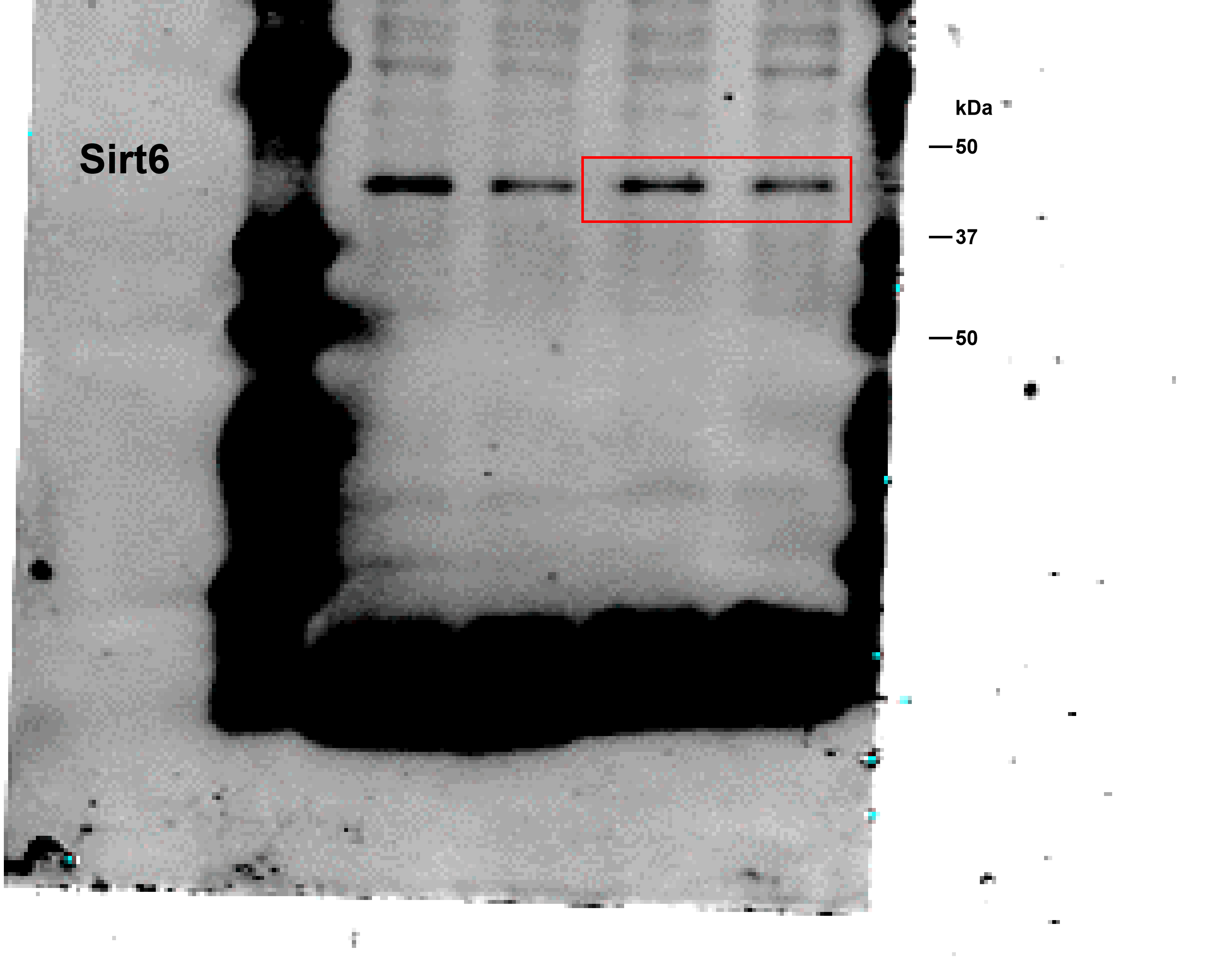

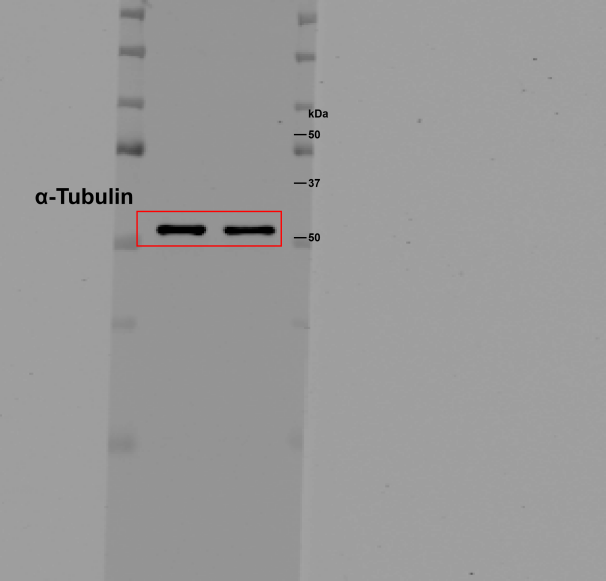


**Figure 2B**


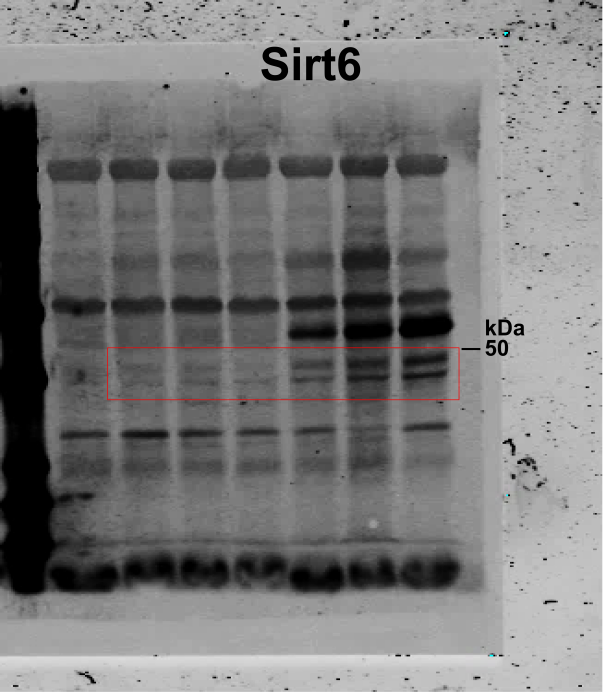

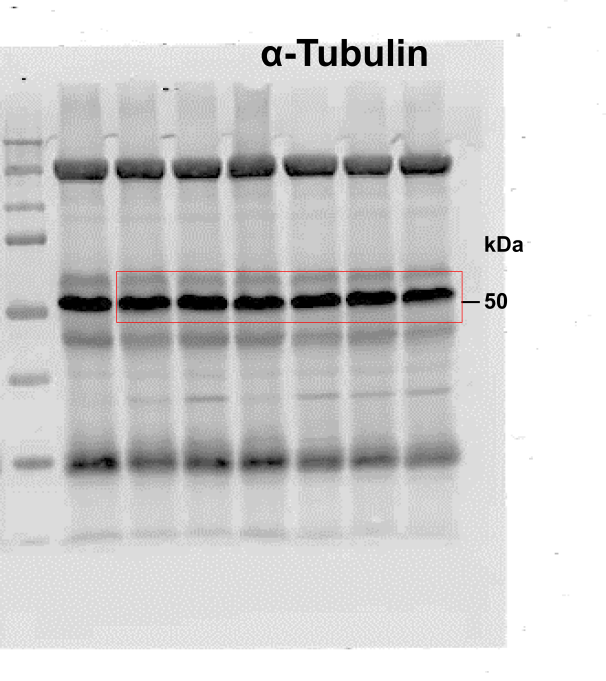


**Figure 3D**


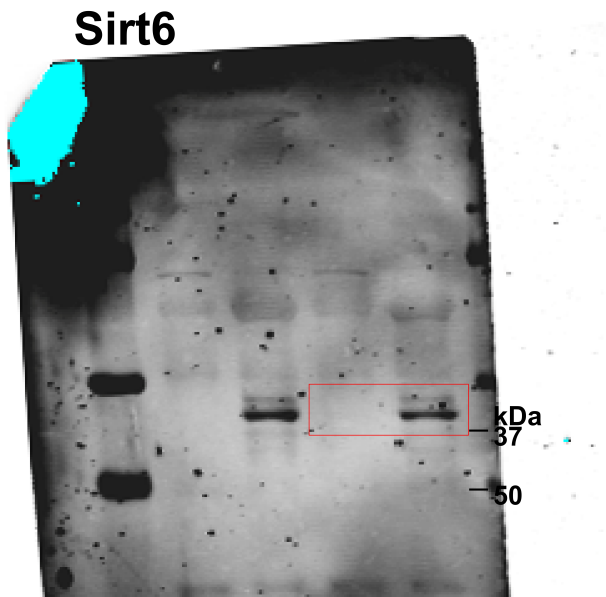

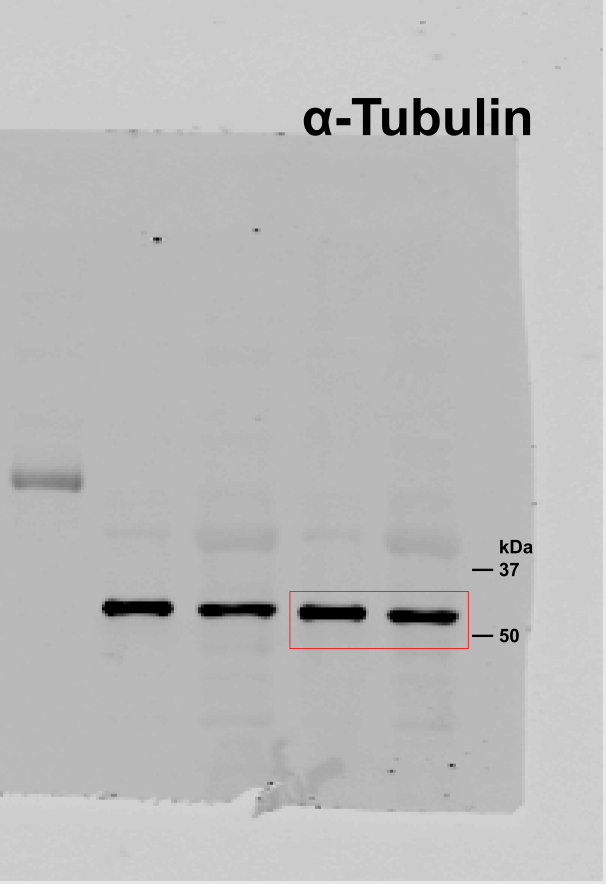


**Figure 4B**


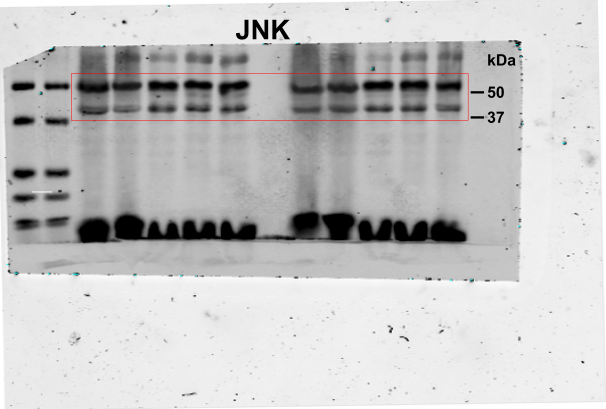

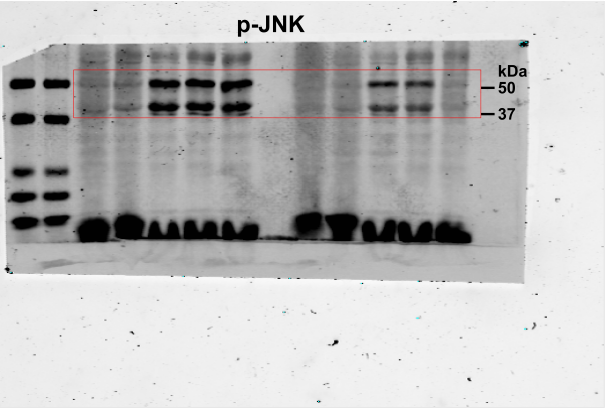


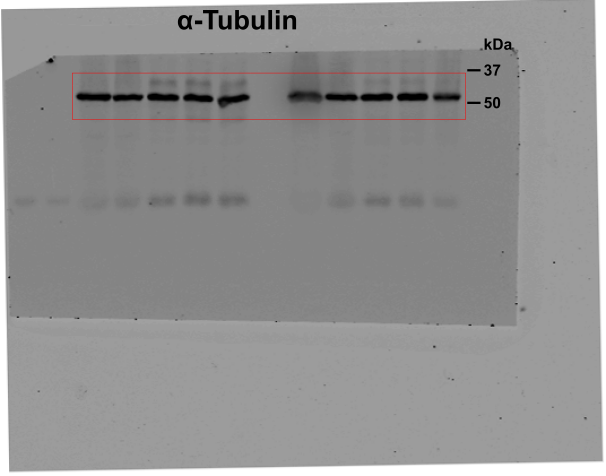


**Figure 4C**


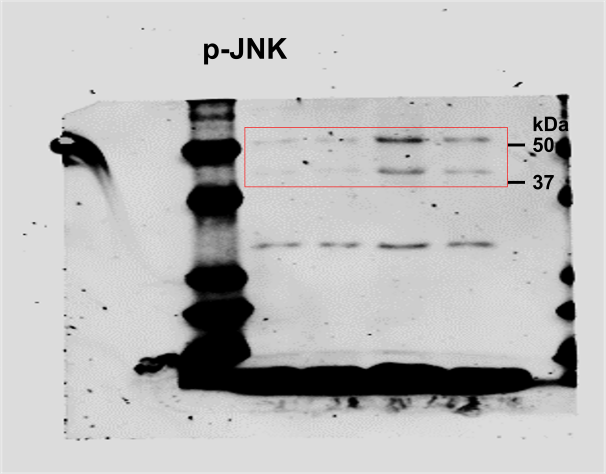

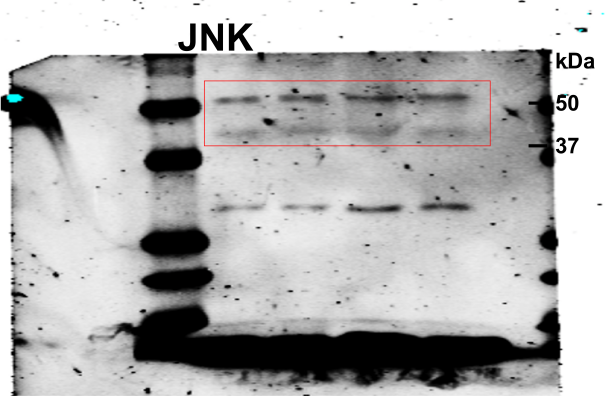

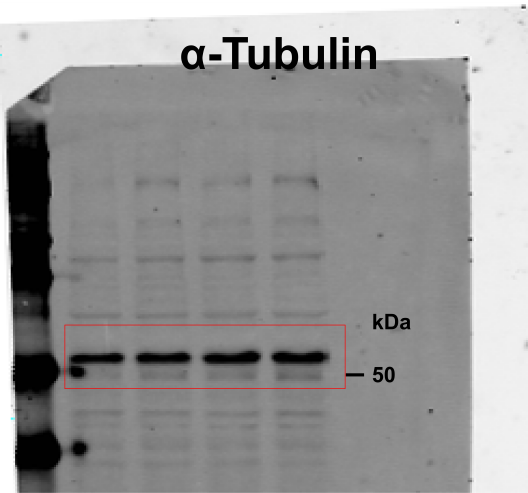


**Figure 4D**


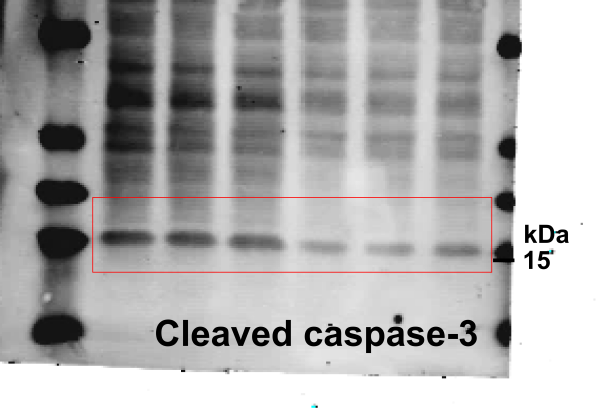

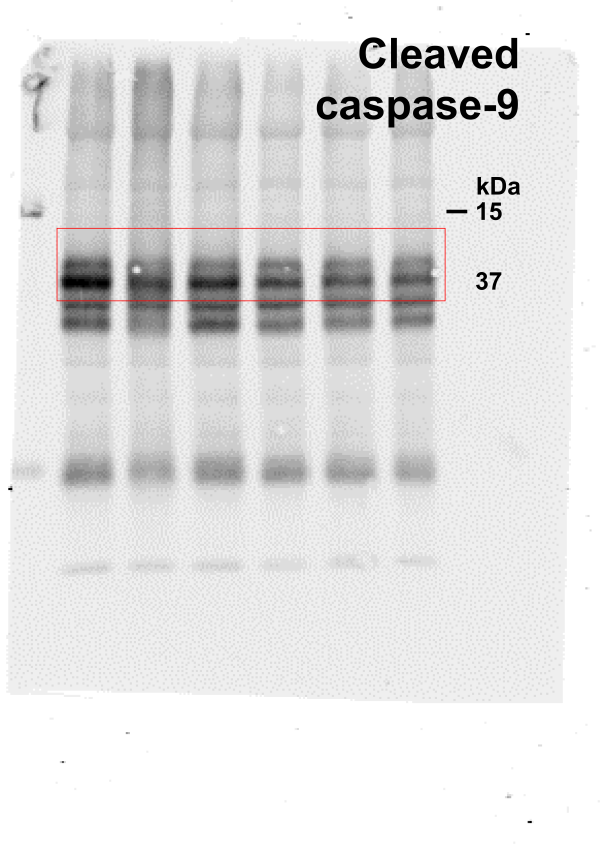


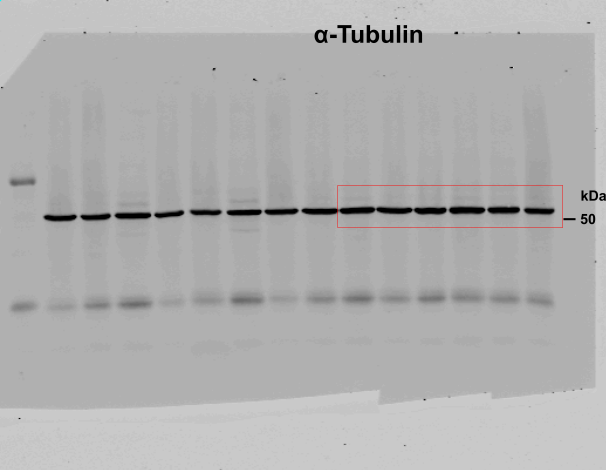


**Figure 4E**


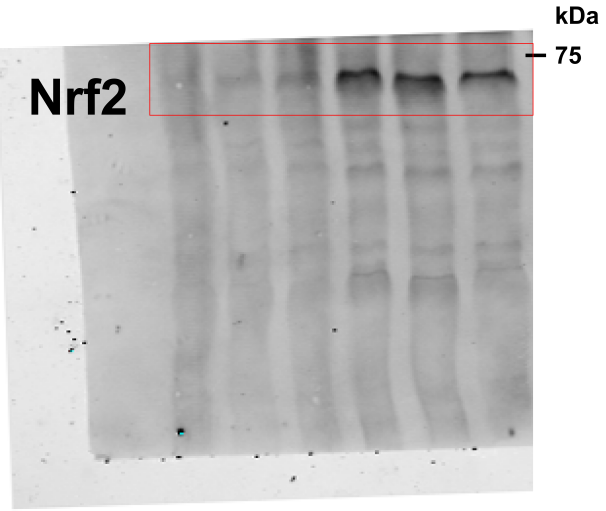

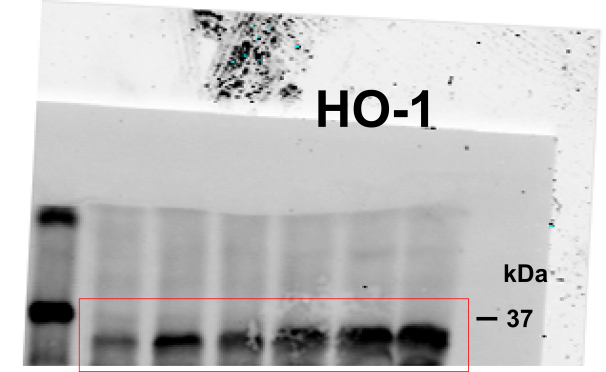


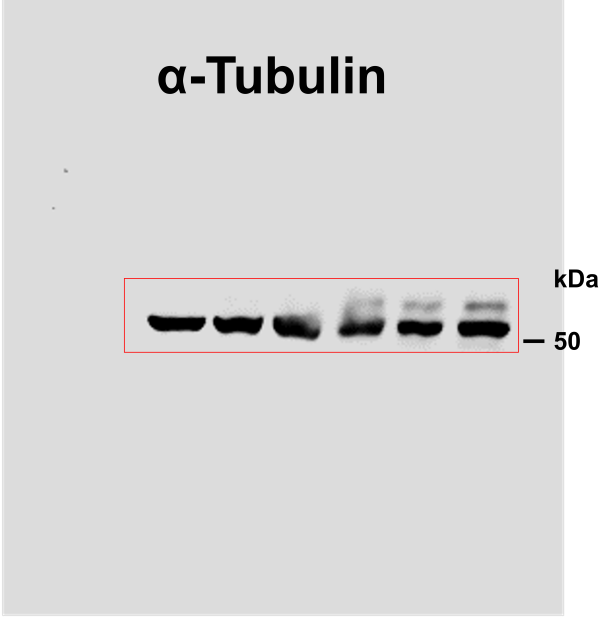


**Figure 4F**


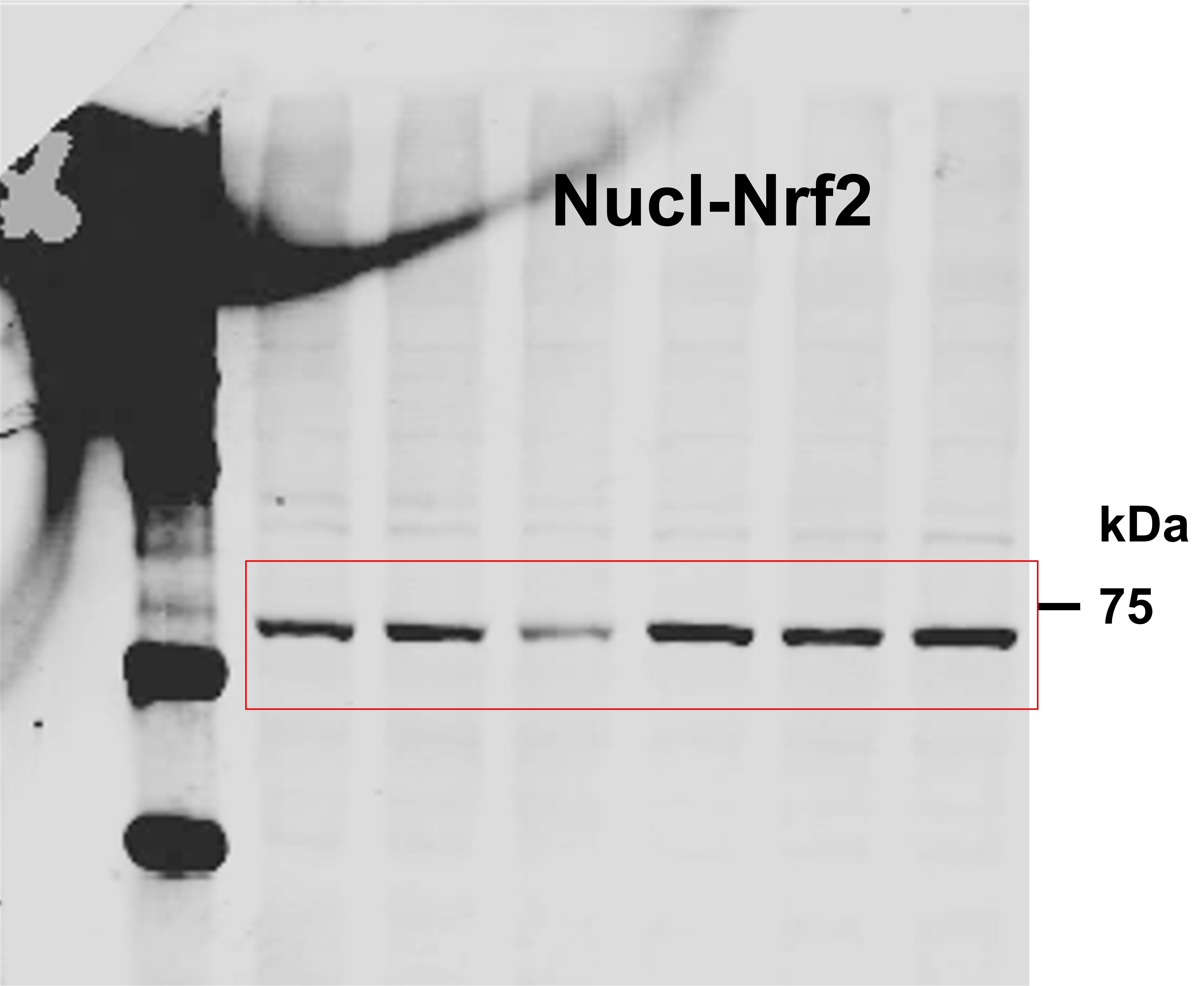

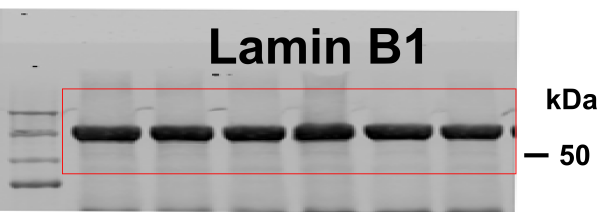


**Figure 5A**


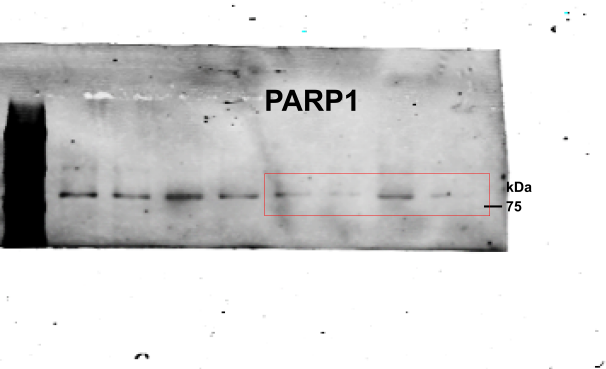

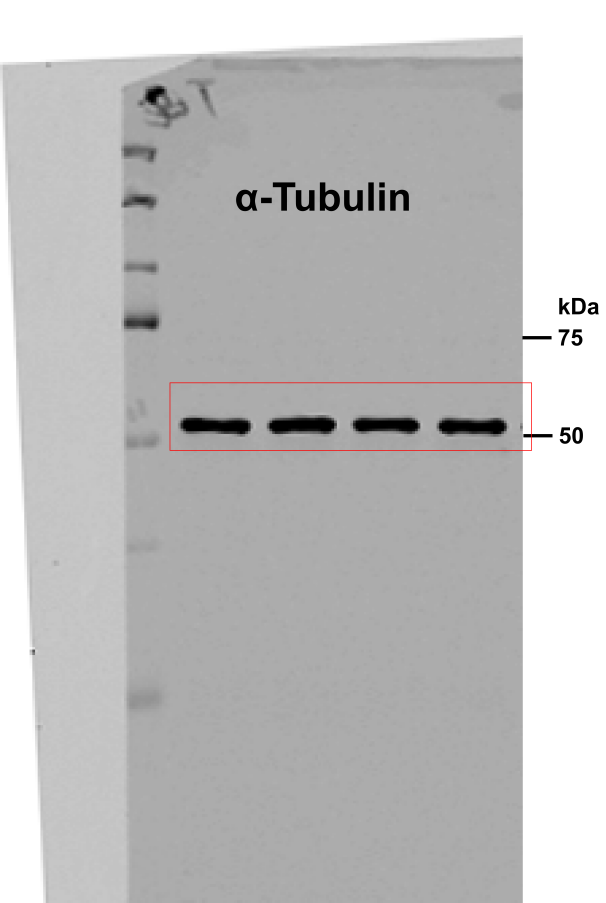


**Figure 5B**


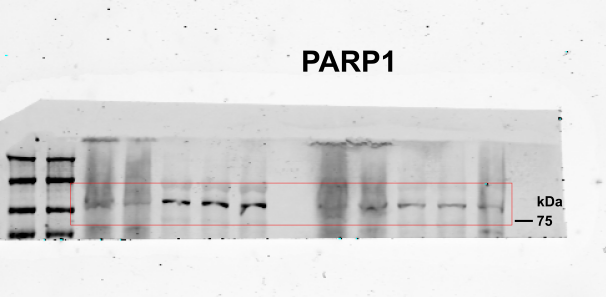

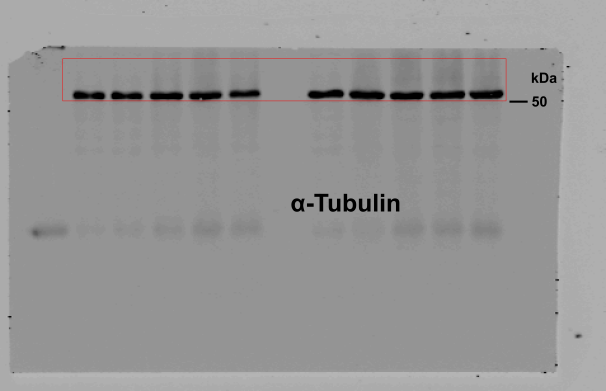


**Figure 5D**

**IP: IgG**


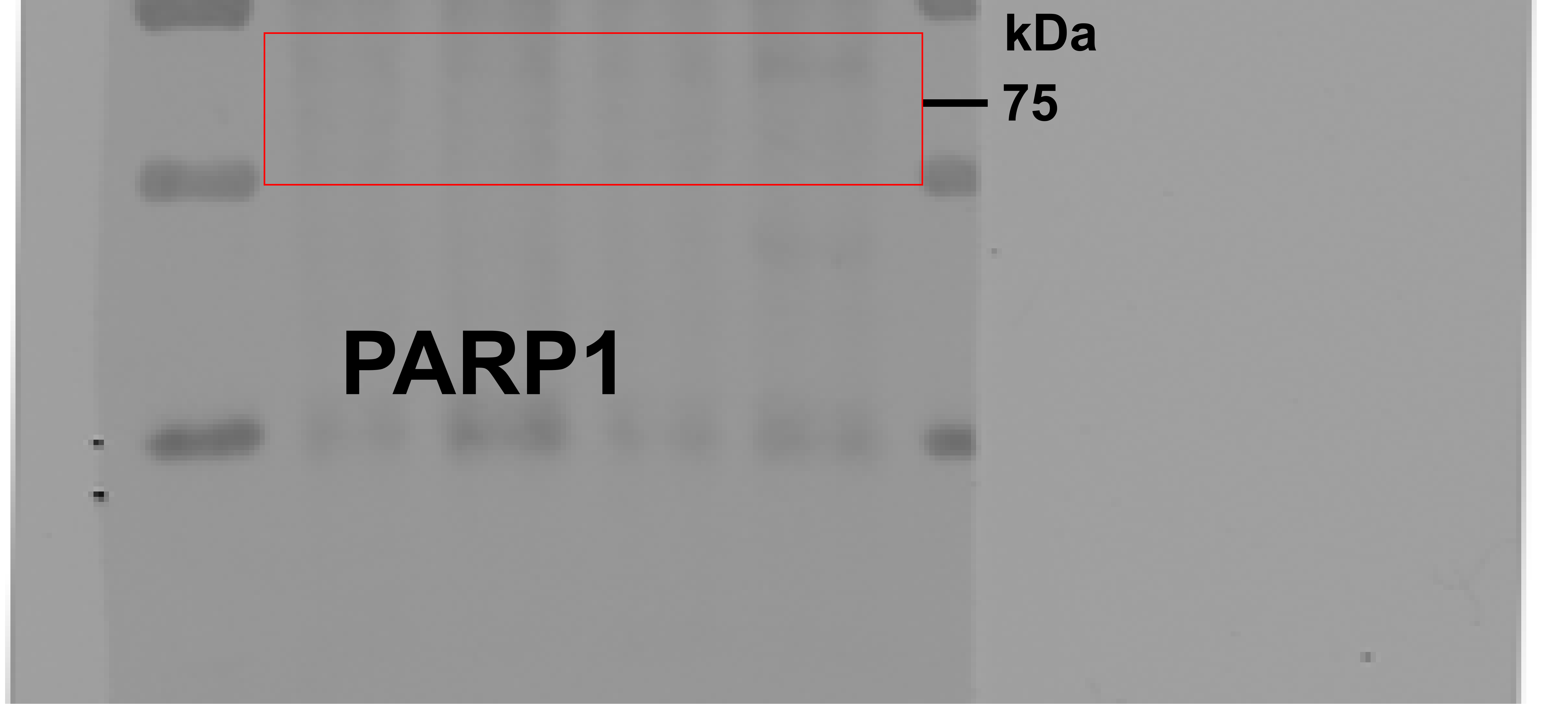

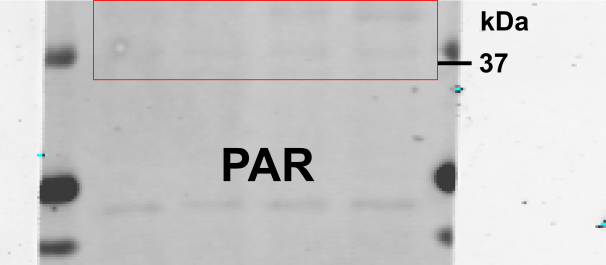


**IP: PARP1**


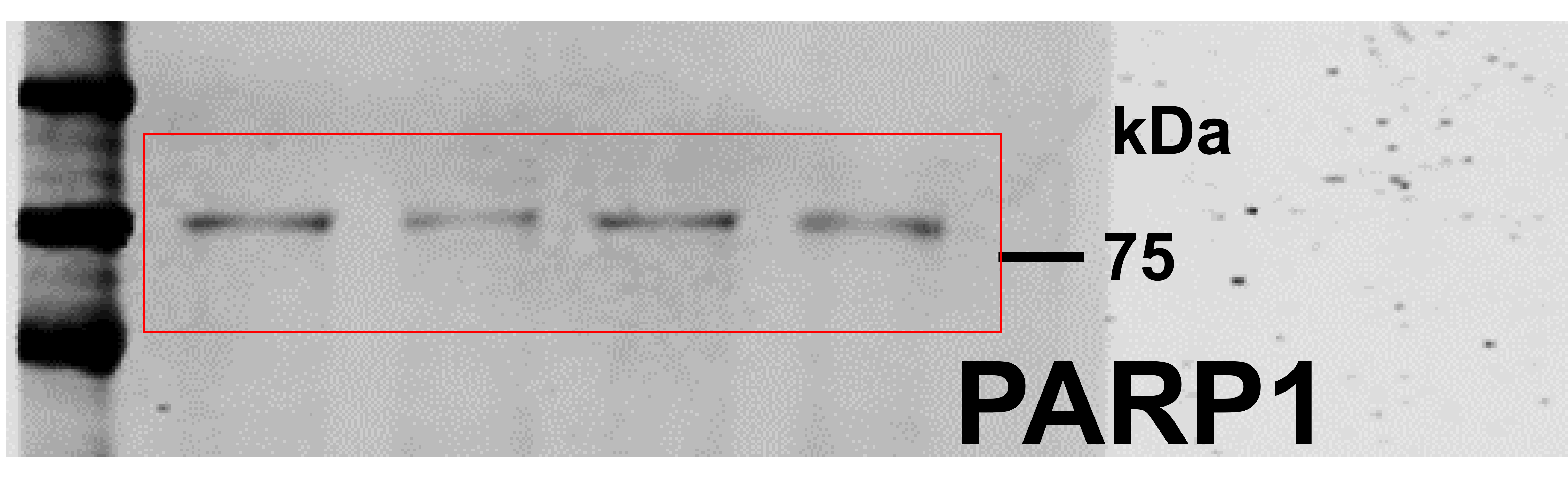

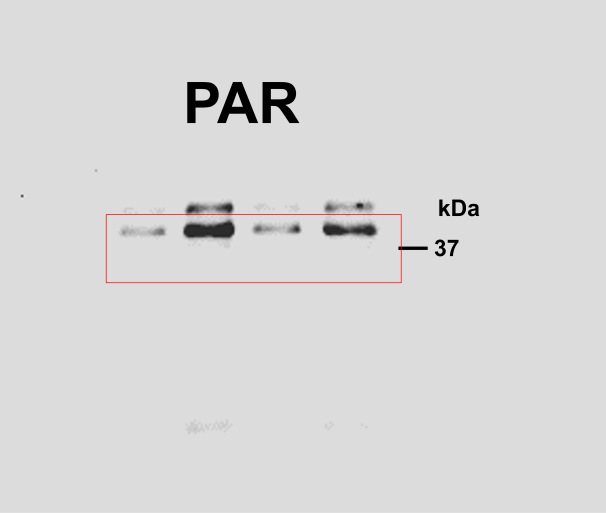


**Input**


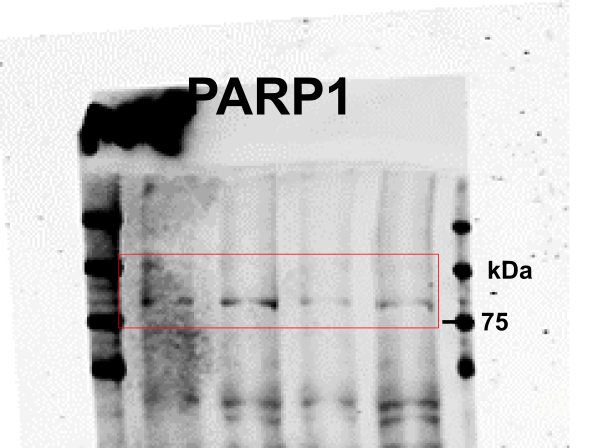

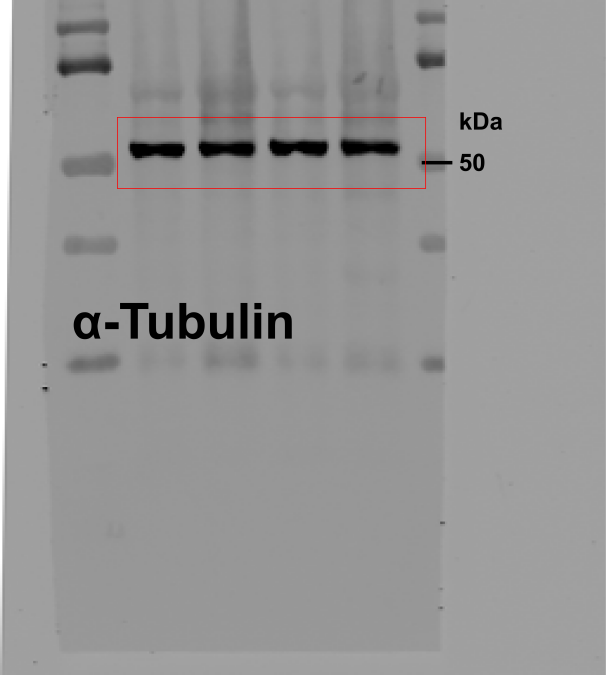


**Figure 7F**


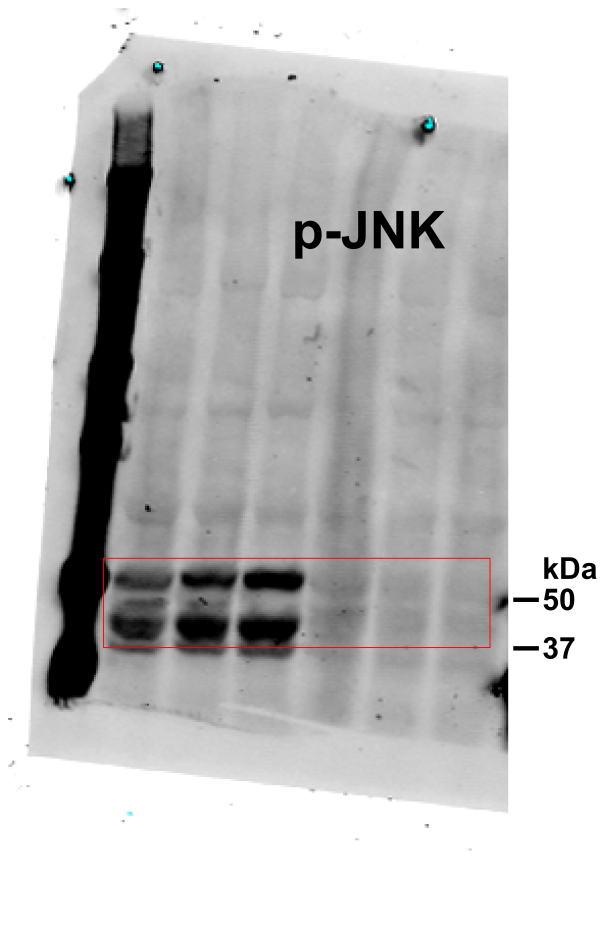

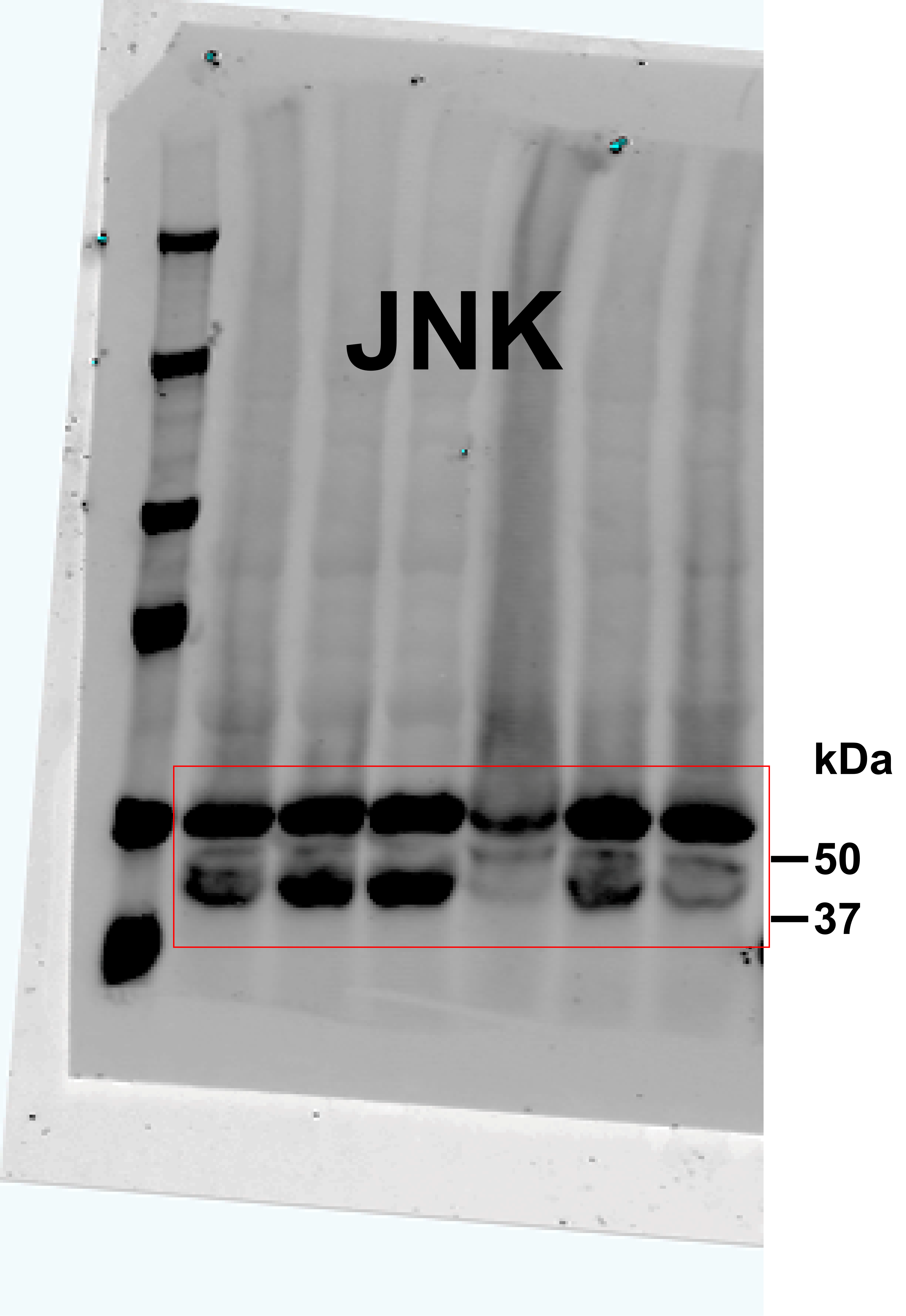


**Figure 8E**


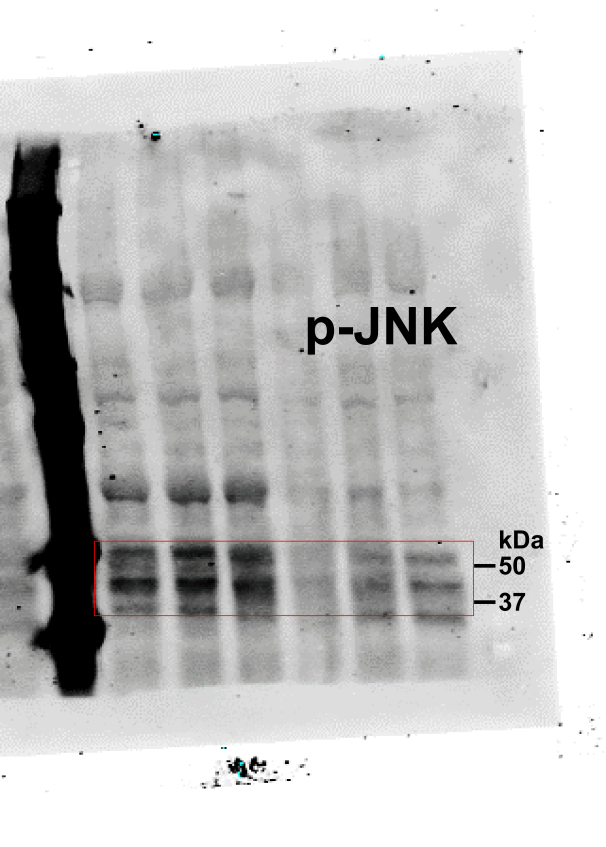

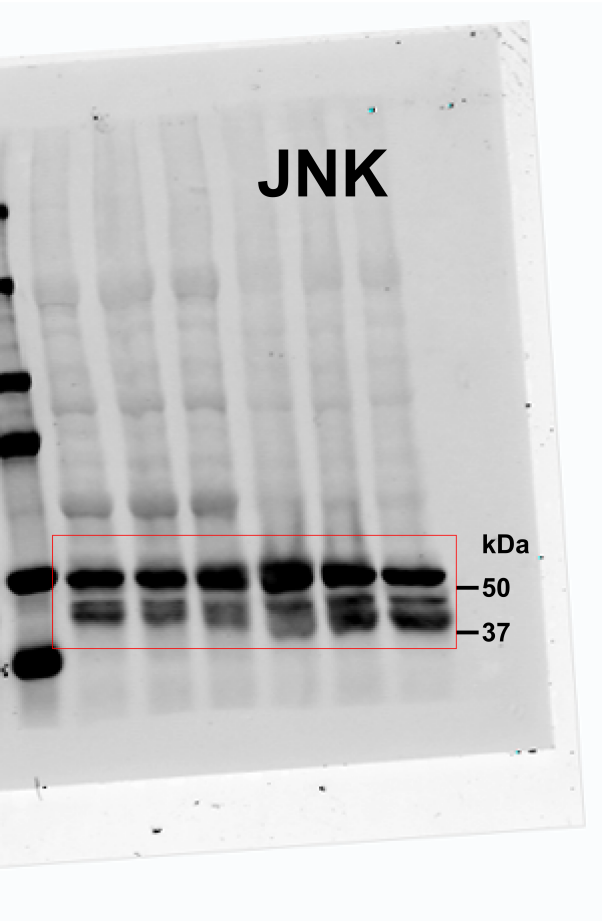

Supplement: Supplementary file 2 — Original Data File [file 41419_2024_6537_MOESM2_ESM.docx]
